# The impact of fabric conditioning products and lint filter pore size on airborne microfiber pollution arising from tumble drying

**DOI:** 10.1371/journal.pone.0265912

**Published:** 2022-04-06

**Authors:** Neil J. Lant, Margaux M. A. Defaye, Andrew J. Smith, Chimdia Kechi-Okafor, John R. Dean, Kelly J. Sheridan

**Affiliations:** 1 Procter & Gamble, Newcastle Innovation Center, Newcastle upon Tyne, United Kingdom; 2 Department of Applied Sciences, Northumbria University, Newcastle upon Tyne, United Kingdom; VIT University, INDIA

## Abstract

Vented tumble dryers release moist warm air from the drying process to the external environment, usually through pipework linking the appliance to a vent in an exterior wall. Although such dryers contain a lint filter to remove fibers from this air stream, recent reports suggest that this process is incomplete, leading to microfibers being released in the ducted warm air and subsequently polluting the external environment. Microfiber release from wash loads comprising 10 100% cotton and 10 100% polyester T-shirts (total load mass ratio 48% cotton, 52% polyester) was measured at different stages of the washing and drying process to compare the quantities of fibers released ‘down the drain’, collected in the dryer lint filter, and released to air from the tumble dryer. Testing under both European and North American washing conditions found that the quantities of microfibers released to air during tumble drying were significant and comparable to levels released ‘down the drain’ during washing. Use of conventional rinse-added liquid fabric conditioner increased microfiber accumulation on the dryer lint filter, with reduced release from the dryer exhaust observed at the highest fabric conditioner dose tested (21.6% and 14.2% reduction under North American and European conditions, respectively). Conventional liquid fabric conditioner did not significantly impact microfiber release from the washing machine, in line with previous studies. A fabric conditioner specially designed for anti-wrinkle performance reduced microfiber release from the dryer exhaust at all levels tested (by 17.6–35.6%, depending on dose), apparently by increasing the efficiency of microfiber accumulation in the lint filter. Tumble dryer sheets were also found to cause a reduction in microfiber release from the dryer exhaust (by 14.1–34.9%, depending on the dose/product), likely driven by collection of liberated fibers on the sheet during the drying process. The use of both antiwrinkle liquid fabric conditioner and dryer sheet enabled a 44.9% reduction in microfiber emissions from the dryer exhaust. In all studies, the fiber mass collected on the lint filter or emitted from the dryer exhaust was richer in cotton fibers (range 83.4–96.3% on the lint filter, 93.0–99.8% from the dryer exhaust) than the wash load composition (48% cotton). Moreover, fibers collected by the lint filter contained a higher proportion of polyester than emissions from the dryer exhaust (range 3.7–16.6% on the lint filter, 0.2–7.0% from the dryer exhaust). There is significant variation in the porosity of lint filters among installed vented tumble dryers. Single-variable testing of the impact of lint filter design concluded that reducing screen pore size significantly reduces airborne microfiber release during tumble drying; a reduction in lint filter pore size from 0.2 mm^2^ to 0.04 mm^2^ reduced release by 34.8%. As some lint filters have pore sizes of around 1 mm^2^, there is enormous scope to reduce microfiber release from dryers though improved lint filter design. However, it is suggested that a step-change in appliance design away from vented dryers to only fully-sealed condenser dryers might be necessary to eliminate the contribution of tumble drying to airborne microfiber pollution.

## Introduction

### Contribution of textile washing to aquatic microfiber pollution

Microplastic pollution has been widely recognized as a significant environmental issue with many implications. Browne et al. [1] reported that textile microfibers arising from laundering of textiles plays an important role in marine microplastic pollution. Since then, many articles have been published relating to the impact of different fiber types, methods of textile construction, fabric care products, washing machine type and cycle choice [2–5]. Synthetic microfibers (e.g. polyester, polyamide, acrylic) are believed to present more of an environmental issue due to their significantly slower rates of biodegradation compared to natural fibers (e.g. cotton and wool) or regenerated fibers produced from natural feedstocks (e.g. rayon) [6, 7]. Several strategies are now being considered to reduce the levels of aquatic microfiber pollution arising from clothes washing [8], including modification of washing machines, modification of washing procedures, modification of textiles and improvements in wastewater treatment plant technology. Selection of specific washing cycles or washing machines [9–11] can significantly reduce the release of microfibers, and various filtration devices have been designed to be used in conjunction with washing machines to reduce microfiber release with mixed results [12, 13]. It is likely that a combination of several approaches will be needed to find an effective long-term solution to aquatic microfiber pollution arising from clothes washing. Approaches that are already attracting interest from legislators include capture by washing machines with built-in filtration devices and improved textiles with lower shedding rates. Such initiatives by official bodies, including a law passed by France requiring new washing machines to be fitted with microfiber filtration devices from 2025, are reviewed by Gaylarde et al. [8]. Vassilenko et al. [14] recently reported an 850-fold difference in the number of microfibers lost between low- and high-shedding textiles, confirming the importance of textile design as part of the solution.

### Non-aquatic environmental microfiber pollution

More recently, several reports have highlighted other non-aquatic types of environmental microfiber pollution arising from textiles, particularly airborne and terrestrial pollution. Dris et al. [15] concluded that atmospheric fallout could pose a significant source of microfiber pollution estimating annual deposition of 3–10 tonnes of fibers onto the Paris conurbation (2,500 km^2^), although not all fibers detected were likely to originate from textiles. Tunayan Kaya et al. [16] used a vacuum filtration device to quantify microfiber pollution at two locations, comparing results with terrestrial contamination at the same sites with strong evidence that textile fibers were present. Beaurepaire et al. recently reviewed 33 published papers relating to atmospheric pollution by microplastics [17]. They concluded that more work was needed to better understand the magnitude and sources of the issue. De Falco et al. [18] first demonstrated that direct microfiber release from clothing to air during wear could be a significant source of microfiber pollution and one of equal importance to aquatic release. Sheridan et al. [19] recently demonstrated the ease to which fibers are able to transfer from person to person through the air with no physical contact. Gavigan et al. [20] estimated that annual release of microfibers to terrestrial environments (141.9 kT yr^-1^) and landfill (34.6 kT yr^-1^) combined are now exceeding release to water bodies (167.2 kT yr^-1^).

### Tumble drying as a source of microfiber pollution

Consumers around the world typically dry their clothes in one of five ways:

- Dried indoors without mechanical action, typically using an airing rack, which may be heated, or a radiator. Dehumidifiers are sometimes used to assist with this process by removing excess moisture from the air.
- Dried outdoors, typically by pegging the textiles to a washing line.
- Using a vented tumble dryer, involving expulsion of warm moist air from the appliance to the outside of the building, with some filtration of air to remove fibers and other debris using a lint filter built into the dryer. These lint filters are designed to be cleaned by consumers after every use with collected fibers disposed in household trash as municipal solid waste. The composition of dryer lint was recently reported by Kannan and Banat [21].
- Using a condenser dryer, involving a sealed system whereby water removed from the fabrics is condensed and collected or drained away, with any fibers generated during the drying process collected by either the lint filter (cleaned in a similar way to vented dryers) or on the condenser. The condenser is also designed to be cleaned by consumers, typically monthly and usually involving water. Therefore, fibers accumulated in dryer condensers could be a source of aquatic microfiber pollution.
- Using the drying function of a combined washer/dryer appliance. Some of these have a lint filter, but many remove any released fibers and moisture through the wastewater pipe.

The incidence of these different drying methods differs around the world with external line drying favored in warmer and dryer climates. However, the perceived convenience of tumble dryers has led to them becoming increasingly popular in countries such as Spain [22]. Tumble dryer use is especially common in the U.S.A. [22], and external line drying is even prohibited by homeowner associations in some areas, although the ‘Right to Dry’ movement has succeeded in making such bans void and unenforceable in some states.

Recently, tumble dryers have emerged as a potentially significant source of microfiber pollution. O’Brien et al. [23] highlighted mechanical drying as a pathway to airborne microplastic pollution, describing studies involving a polyester fleece blanket tumble dried in an enclosed room. Tumble drying caused a significant increase in fiber levels detected in the room through air sampling. Kapp and Miller [24] conducted experiments using externally vented tumble dryers, elegantly measuring fiber deposition onto snow in the area surrounding the dryer vent to quantify emission levels from polyester fleece blankets. This confirmed a direct link between microfiber emissions from vented dryers and terrestrial microplastic pollution. Further studies describing microfiber release from tumble dryers have been reported by Kärkkäinen and Sillanpää [25]. However, these were focused on fiber collection by the lint filter, and it was not clear whether they used a vented or condenser dryer as the specific dryer model number was not given, only that it was from the Bosch Series 4 range of appliances which includes both types of dryer. A very recent report [26] concluded that tumble dryers release 433,128–561,810 microfibers to air during 15 minutes of use corresponding to an annual release of between 9 x 10^7^ to 12 x 10^7^ microfibers from an average Canadian household. This was reported to be greater than the quantity of fibers released by washing machines, although no actual comparison is made with the same fabric load. A powerful tumble dryer (Electrolux^®^ Wascator^®^ TT200) similar to those used in commercial laundries was used, raising concerns that the results might not correlate with domestic dryers. Moreover, the studies did not consider the relevance of the built-in lint filter of these dryers which is designed to collect fibers from the exhaust and be cleaned regularly by the user.

The first objective of the present study is to compare, for the first time, the levels of microfibers released during washing and tumble drying of the same wash load. A second objective is to evaluate the impact of rinse-added (liquid fabric conditioners) and dryer-added (dryer sheets) fabric care consumer products on these phenomena, building on our previous studies focused on release from washing machines [9–11]. The third objective is to evaluate the impact of lint filter design on airborne microfiber release from tumble dryers as a potential approach to mitigate the issue. The findings will be relevant to those involved in microfiber research within the textile and appliance industries, to companies involved in the development and manufacture of fabric care products, and to government and related organizations considering legislation aimed at reducing microfiber pollution.

## Materials and methods

### Textiles

Each load comprised 10 cotton T-shirts (Fruit of the Loom^®^ Original T-shirt, product code 61–082, size L, 100% cotton, density 145 g/m^2^) and 10 polyester T-shirts (Fruit of the Loom^®^ Performance T-shirt, product code 61–390, size L, 100% polyester, density 140 g/m^2^). Due to the mass differences between the cotton and polyester T-shirts, these loads comprise 48% cotton fibers and 52% polyester fibers. The garments were new and unused and the color and supplier for each test are listed in S1 Table. Loads were weighed before the first washing to enable calculation of microfiber release as a proportion of load mass.

### Fabric care products

All tests with European washing conditions were conducted using one Ariel^®^ 3in1 detergent pod per wash as the detergent. Tests with North American washing conditions were conducted using one Tide^®^ 3in1 detergent pod per wash as the detergent.

The North American conventional liquid fabric conditioner test was conducted using Ultra Downy^®^ April Fresh with 50.5 g as the recommended dose. The European conventional liquid fabric conditioner test was conducted using Lenor^®^ Spring Awakening with 25 mL as the recommended dose. The North America antiwrinkle liquid fabric conditioner test was conducted with Downy^®^ Wrinkle Guard Fresh test with 60 g as the recommended dose. These three tests were conducted with the following four dosages of fabric conditioner: Nil fabric conditioner, single dose, 1.5x dose and double the recommended dose. In all cases, liquid fabric conditioner was dosed into the dedicated conditioner compartment of the washing machine where it is automatically released into the machine during the final rinse cycle.

The North American dryer sheet test was conducted using Bounce^®^ Outdoor Fresh dryer sheets and Bounce^®^ WrinkleGuard Mega dryer sheets. Impact of dryer sheets on microfiber release was conducted with the following four treatments: Nil dryer sheet, 1 Bounce^®^ Outdoor fresh dryer sheet, 3 Bounce^®^ Outdoor fresh dryer sheets and 1 Bounce^®^ WrinkleGuard Mega dryer sheet. Dryer sheets were always added to the tumble dryer with the damp clothing at the start of the drying cycle and are removed and discarded at the end when the dry fabrics are removed.

The North American test involving a combined system of antiwrinkle liquid fabric conditioner and dryer sheet was conducted using the following two treatments: Nil dryer sheet/antiwrinkle liquid fabric conditioner, and a combination of 120 g Downy^®^ Wrinkle Guard Fresh (i.e. double recommended dose, added to the conditioner compartment) and one Bounce^®^ WrinkleGuard Mega Dryer Sheet, added to the tumble dryer.

All fabric care products were manufactured by Procter & Gamble. European and North American products were sourced from retailers in the U.K and U.S.A., respectively, during summer 2020.

### Washing and drying procedure

#### North American washing procedure

All North America testing, i.e. liquid fabric conditioner test, antiwrinkle liquid fabric conditioner test, dryer sheet test and the test involving a combined system of antiwrinkle liquid fabric conditioner with dryer sheet were conducted with typical North American washing conditions. Tests were conducted using 6 grains per U.S. gallon hardness water and a High Efficiency top-loader washing machine Maytag^®^ Bravo (Model MVWX655DW1). Tests were conducted using the customized North America Median program (Cycle settings: Medium soil, Fabric Conditioner knob set to “ON”, Extra rinse knob set to “OFF”, Washing temperature: 25°C, Main wash volume: 38 L, Rinse temperature: 15°C, Rinse volume: 43 L, and 52 minutes total duration). The test garments were washed and rinsed using these conditions, and the same conditions were used for a washout cycle afterwards conducted without fabrics and products to clean the machine. In tests involving measurement of ‘down the drain’ microfiber release, fibers were collected and combined from both the clothing wash/rinse process and the washout cycle used to clean the appliance. A further washout cycle was conducted without fiber collection prior to the appliance being used again. All North American washing machine tests were conducted using sets of four identical machines fed by the same water supply. Treatments were rotated between the four washing machines and conducted in triplicate for four consecutive washing cycles. I.e., each test involved the test loads being washed and dried four times.

#### European washing procedure

The European liquid fabric conditioner test and lint filter pore size test were conducted with European washing conditions. Both tests were conducted using 19 grains per U.S. gallon hardness water and Miele^®^ W3622 washing machines using the 30°C Cotton Short program (85 minutes total duration, 1600 rpm spin speed) as washing cycle and the 40°C Express Wash program (30 minutes total duration, 1600 rpm spin speed) as the washout cycle conducted without fabrics or products. As with the North America tests, where ‘down the drain’ microfiber release was measured, fibers were collected and combined from both the clothing wash/rinse process and the washout cycle used to clean the appliance. A further washout cycle was conducted without fiber collection prior to the appliance being used again. All European washing machine tests were conducted using a set of four identical machines fed by the same water supply. Treatments were rotated between the four washing machines and conducted in triplicate for 4 cycles, in line with the North American testing.

#### Drying procedure

Vented tumble drying was conducted in the same way for all tests using the same appliance model. Loads were dried after each cycle using Indesit^®^ vented tumble dryers (model IDV75) for one hour on the high heat setting. Mechanical and thermal energy settings were the same for each load. A set of two identical dryers was used and treatments were rotated between the two machines. The tumble dryers were placed on a balance to measure the drying process in real time, located in a specialized laboratory for appliance research with high levels of ventilation. Internal temperature (using a probe) and power consumption (at the power supply outlet) were recorded during drying to ensure good consistency between loads. The measurement of dryer mass during the drying process confirmed that loads were completely dry after 1 hour.

### Microfiber collection

#### Collection during the washing cycle

Microfibers were collected from washing machine effluent and quantified based on the general approach described by Napper and Thompson [27], as used by Kelly et al. [9] and Lant et al. [10]. Thus, evaluation of the impact of European and North American conventional liquid fabric conditioners and North American antiwrinkle liquid fabric conditioner on microfiber release ‘down the drain’ were conducted by collecting the wastewater for the first and the fourth cycles. Any fibers remaining in the machine were washed out and collected by running an additional cycle without any garment and product, as described in the washing procedures. Water was collected into 25 L high density polyethylene containers from the drain hose of the washing machine. Microfibers were collected by filtration of all wash, rinse and washout water through a 20 μm CellMicroSieve^®^ (BioDesign Inc., Carmel, N.Y., U.S.A.). At the end of the filtration, the nylon mesh was thoroughly washed with clean water to re-suspend the collected fibers.

#### Collection during the drying cycle

**Fig 1:**
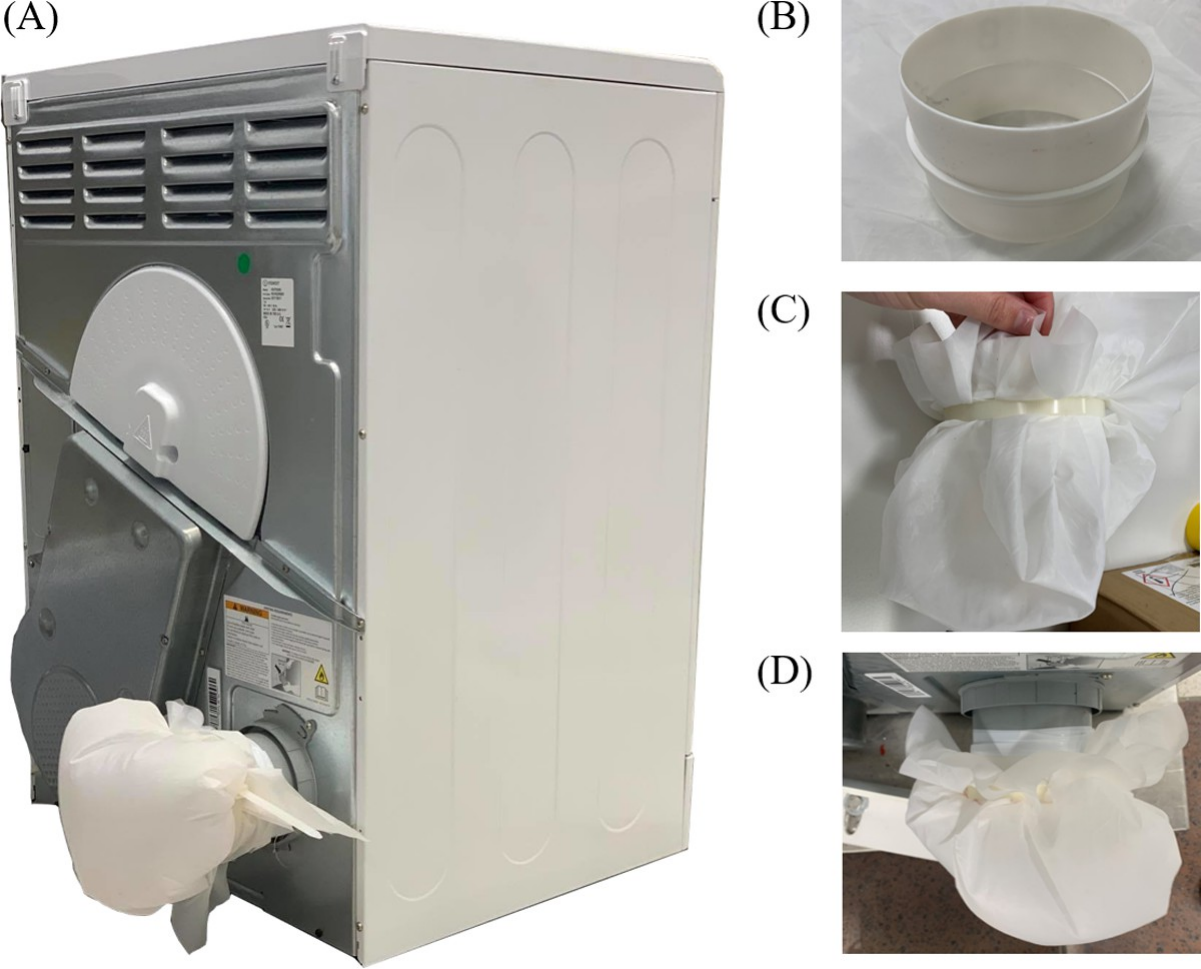
Tumble dryer images. Rear of tumble dryer **(A**) with 20 μm CellMicroSieve^®^ installed to collect microfibers from the exhaust. The CellMicroSieve^®^ is secured to a 100 mm plastic pipe connector (**B**) using a cable tie as shown in (**C**). The pipe connecter was then secured to the dryer exhaust pipe using electrical tape as shown in (**D**).

Evaluations of the microfiber release to the dryer exhaust were conducted by collecting the microfiber at the air exit for each of the four cycles in all tests, as illustrated in Fig 1A. Microfibers were collected using 20 μm CellMicroSieve^®^ (BioDesign Inc., Carmel, N.Y., U.S.A.), attached to the dryer exhaust using a 100 mm plastic pipe connector (Fig 1B) (model 414c, Manrose Manufacturing Ltd., U.K.). The CellMicroSieve^®^ was connected to one side of the plastic pipe connector using 450 mm long, 10 mm wide cable ties (product 90526, Screwfix Direct Ltd., U.K.) as shown in Fig 1C and then connected to the vent pipe using electrical tape as shown in Fig 1D. The addition of the mesh at the end of the air exit did not impact the air flow of 12 L/min. At the end of the drying, the CellMicroSieve^®^ was thoroughly washed with clean water to re-suspend the collected fibers.

Microfibers caught in the lint filter were collected for the four cycles. The tumble dryer lint filter used in all experiments, except one leg of the test evaluating impact of lint filter design, was the original lint filter from the dryer Indesit^®^ IDV75 with a pore size of 0.2 mm^2^. At the end of the drying cycle, the nylon mesh was washed with clean water to suspend the collected microfibers.

**Fig 2:**
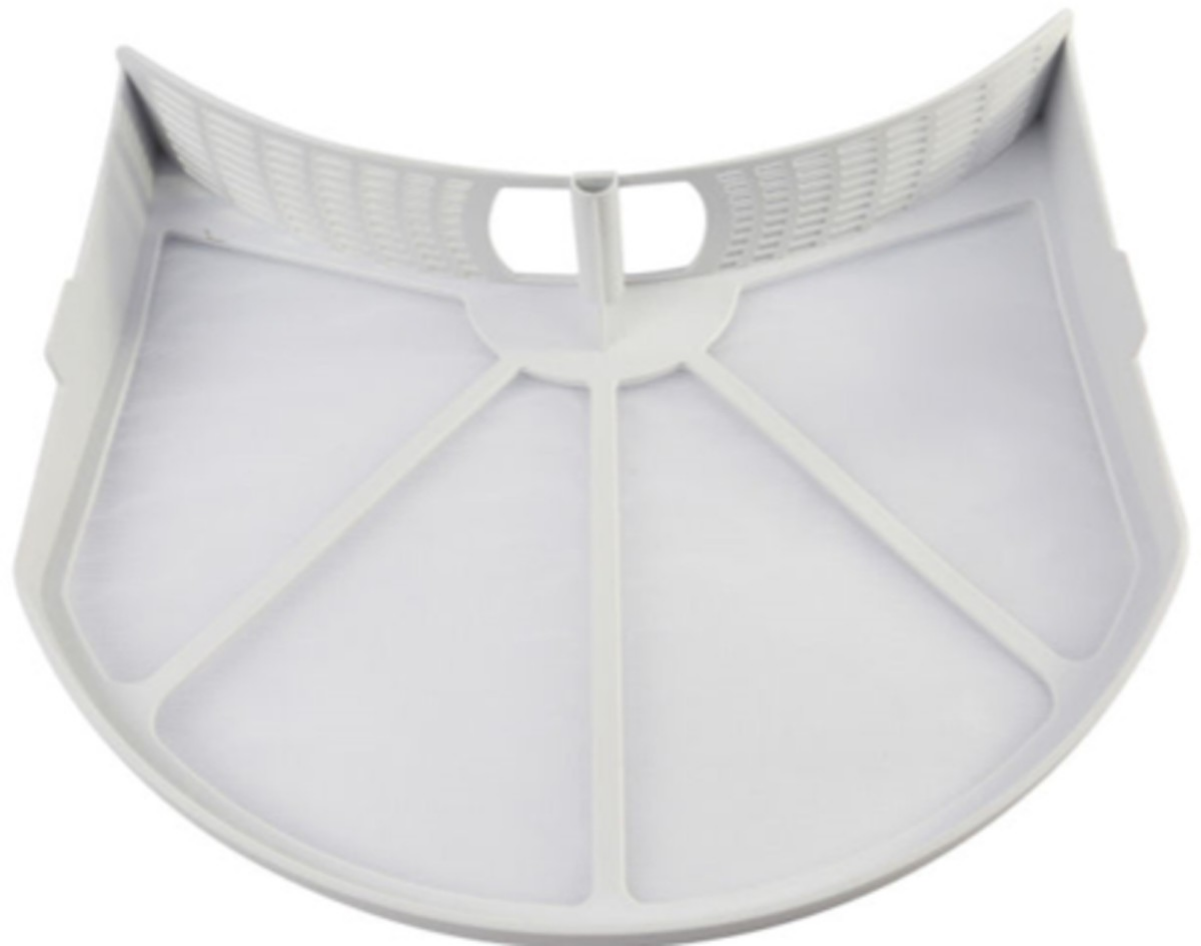
Lint filter device from Indesit tumble dryer. Lint filter component as purchased. During the drying cycle, fibers accumulate on the white mesh regions which are supported by a plastic frame.

**Fig 3:**
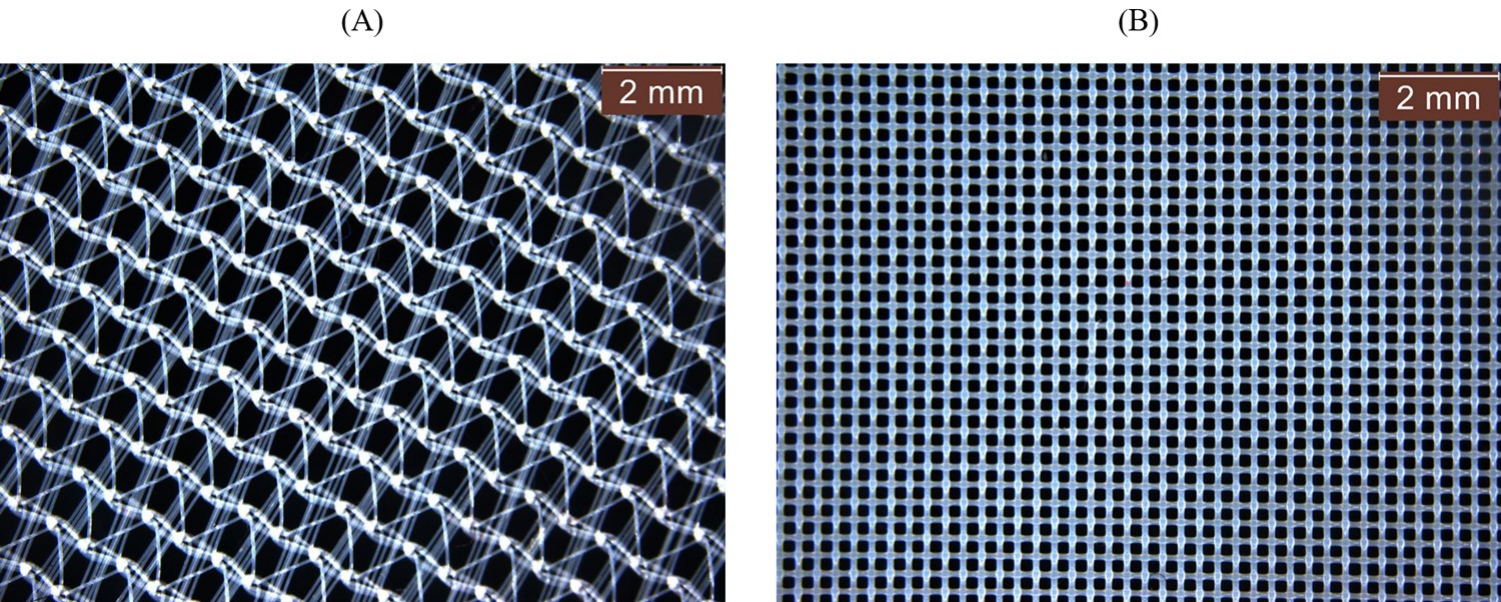
Lint filter mesh. Microscopy images of the mesh from the relatively coarse Indesit^®^ (**A**) and fine Miele^®^ (**B**) lint filters used to compare impact of lint filter pore size on microfiber collection.

Different mesh sizes are used by the dryer manufacturers. Evaluation of the impact of the lint filter on the microfiber release was conducted by using two different lint filter pore sizes; the relatively coarse lint filter supplied with the appliance and a fine lint filter created by replacing the mesh in the original filter. The original lint filter of the Indesit^®^ Dryer (see Fig 2) used as a coarse lint filter has a pore size of 0.2 mm^2^. To create a lint filter for the same dryer with a finer pore size, additional Indesit lint filters were purchased, and their mesh replaced with that removed from Miele^®^ Tumble dryer lint filters (Miele^®^ part number 6244611) which have a pore size of 0.04 mm^2^. Loctite^®^ All Plastic Super Glue (Henkel Ltd., U.K.) was used to secure the Miele^®^ mesh onto the Indesit^®^ lint filter frame, maintaining the same filtration surface area of 270 cm^2^. Fig 3 uses light microscopy images to compare the relatively coarse Indesit^®^ (Fig 3A) and fine Miele^®^ (Fig 3B) lint filter mesh used for these tests. Vented dryers were cleaned before each drying cycle to remove remaining microfiber(s) by running a 5-minute cycle without any fabric, filter or product inside.

#### Mass measurement

Microfiber solution was filtrated onto pre-weighed Whatman^®^ No 541 filter paper (G.E. Life Sciences, Little Chalfont, U.K.) using a Büchner funnel under vacuum before drying overnight at 50°C.

The mass of collected fibers was then calculated, corrected for the percentage loss in filter paper weight on drying which was determined by recording the mean percentage mass loss on drying of 10 similar papers. Microfiber release data are presented in parts per million (ppm), i.e. as mg of released fiber per kg initial dry fabric load mass. Statistical significance was determined using Student’s t-test; comparisons with a p-value of <0.050 were judged to be significantly different at 95% confidence level.

## Microfiber composition using chemical method

### Sample preparation

The weight of microfibers collected from either lint filter or dryer exhaust following the fourth drying cycle were determined and recorded. An empty, covered, glass Petri dish was weighed using a balance accurate to 0.1 mg (Sartorius AX124, Germany) and recorded. The lint was then placed into the Petri dish, covered, weighed, and recorded. The weight of the lint was determined by calculating the difference of the two weights.

### Acid digestion of cellulosic microfibers

The method used in determining the composition of cotton to polyester in the weighed samples described in the sample preparation section above was adapted from Chemical Test Method No. 5 of Test Methods D629 [28].

The weighed sample was placed into a 100 mL beaker and covered with 50 mL of 70% H_2_SO_4_ (Analytical Reagent Grade, Fisher Scientific, U.K.) using a 50 mL ± 0.05 mL pipette (Volac®, U.K). This was allowed to stand for 15 minutes at a room temperature of 15 to 30°C, stirring every 5 minutes. The liquid was decanted through a 560 μm stainless-steel strainer (VWR® Test Sieve BS ISO 3310–1, Germany), the sample was returned to the beaker and 50 mL of 70% H_2_SO_4_ was added. The sample remained in the acid for approximately 30 minutes and stirred every 5 minutes. The liquid was strained using a 180 μm stainless-steel sieve (VWR® Test Sieve BS ISO 3310–1, Germany) and the remnant collected on the sieve was washed under running de-ionized water between 1 to 5 minutes. Following this, the sample was covered with 2% NaHCO_3_ (Laboratory Reagent Grade, Fisher Scientific, U.K.) and allowed to stand for at least 5 minutes, then washed with de-ionized water as above.

The residue was collected with a pair of stainless-steel tweezers and blot dried on white paper towels. Caution was applied to avoid loss of loose fibers by visually examining the white paper towels.

The blot dried residue was placed in an open weighing container (glass Petri dish from above) and oven (UM 200 Memmert, Germany) and dried at 105–110°C for a minimum of 1.5 h. The weighing container was removed from the oven immediately and placed uncovered into a desiccator over CaSO_4_ and allowed to cool, in the sealed desiccator, for at least 30 minutes. The Petri dish cover was placed on the sample container, removed from the desiccator, and weighed to the nearest 0.1 mg.

### Sheddability test on selected garments

The properties of textile materials affects their sheddability, i.e., their potential to lose fibers through normal wear and other activities such as laundering and drying. In order to determine the sheddability of the cotton T-shirts and the polyester performance T-shirts used in the washing-drying procedure, the ‘press and rub’ method was used [29]. Firstly, any fibers extraneous to the garment itself present on its surface were removed from the testing area to avoid erroneous counting. An adhesive tape (TapeIt™, 3L Office, Denmark) was applied to the surface of the garment, pressed down and removed, taking with it extraneous fibers from the surface. A fresh adhesive tape (TapeIt™, 3L Office, Denmark) measuring 17 cm by 5 cm was then placed over the same now ‘blanked’ area of the garment. The end of the tape was pressed down onto the surface and using a forefinger, rubbed along its length once. The tape was removed and secured to a clear acetate sheet for more detailed examination. The tape was sectioned into 1 cm^2^ squares and the number of fibers within one randomly selected square was counted with the aid of microscopy (Leica S6 E Greenough stereomicroscope, Leica Microsystems, Germany). This process was repeated six times across the front of the garment to account for variation in pressure whilst doing the ‘press and rub’, and the average number of fibers measured per 1cm^2^ sample calculated.

### Measurement of fibers

Fibers were recovered from tape lifts generated from the sheddability test described in the previous section. Using a low power microscope (Leica Microsystems, Germany), target fibers were identified and marked. Incisions were made on the marked areas of the tape lifts, sticky stuff remover (De-Solv-it®, United Kingdom) applied and using a pair of stainless-steel tweezers (EM-Tec, 5.AM, Switzerland) fibers were removed and mounted individually on glass slides (CIMED^®^, 1–1.2 mm thick, 25 x 75 mm) using glycerol (VWR^®^ CAS number: 56-81-5) and covered with round cover slips (9 mm, Thermo Scientific^®^, Germany). Using an Olympus CX22 microscope (J.B Microscopes Ltd., U.K.) coupled with Euromex camera with Image Focus 4.0 software, measurements of fiber length and width were taken following calibration of the system in the appropriate units (e.g. mm). Twenty randomly selected fibers from a sheddability tape taken from each garment were measured.

## Results and discussion

### Impact of fabric care products

#### Liquid fabric conditioner: North America conditions

North America testing involved detergent (Tide^®^ pods), liquid fabric conditioner (Downy^®^) and typical washing conditions for the region. Microfiber release was measured at three different points: ‘down the drain’ release from the washing machine (recorded in cycles 1 and 4), accumulation in the lint filter of the dryer (cycles 1–4) and emitted from the air exhaust of the dryer (cycles 1–4). A summary of data is given in Table 1 with full data presented in S2 Table. Table 1 shows the average microfiber release at each of the three measurement points for each of the four treatments including a ‘nil fabric conditioner’ control involving washing in detergent only and three different doses of fabric conditioner. Different doses of fabric conditioner were tested to reflect the reality that users of this product do not always fully comply with the standard dosages recommended, and products typically recommend different dosages depending on the level of desired fabric softness. For the test legs involving different doses of fabric conditioner, the percentage change in microfiber release compared to the nil fabric conditioner control is also presented. Results that are statistically different versus the nil fabric conditioner control are denoted by the letter ‘s’ after the percentage difference.

**Table 1:** Summary of microfiber release and fiber analysis data–North America liquid fabric conditioner testing.

	Nil fabric conditioner	Single dose fabric conditioner	1.5 dose fabric conditioner	Double dose fabric conditioner
**Microfiber release**	Average ppm	Average ppm	Average ppm	Average ppm
	Reference	(% change vs Reference)	(% change vs Reference)	(% change vs Reference)
**Down the drain**	63.80	59.03	58.84	76.45
Cycles 1 and 4		(-7.5)	(-7.8)	(+19.8)
**Dryer lint**	106.04	**127.24**	**138.45**	**137.75**
Cycles 1–4		**(+20.0s)**	**(+30.6s)**	**(+29.9s)**
**Dryer exhaust**	50.25	51.70	44.12	**39.38**
Cycles 1–4		(+2.9)	(-12.2)	**(-21.6s)**
**Fiber analysis**	Cotton/Polyester	Cotton/Polyester	Cotton/Polyester	Cotton/Polyester
	%	%	%	%
**Dryer lint**	91.3/8.7	88.7/11.3	87.5/12.5	92.3/7.7
Cycle 4				
**Dryer exhaust**	96.3/3.7	96.5/3.5	95.5/4.5	97.9/2.1
Cycle 4				

The data shows that fabric conditioner significantly increases microfiber collection at the dryer lint filter by 20.0–30.6% but has no significant impact on ‘down the drain’ release from the washing machine. Only the double dose of fabric conditioner has a significant impact on microfiber release from the dryer exhaust, showing a 21.6% reduction compared to the nil fabric conditioner control. These results are consistent with Lant et al. [10] in showing no significant impact of fabric softener on ‘down the drain’ microfiber release and suggest that fabric conditioner promotes microfiber accumulation at the lint filter. As these fibers should be disposed in household trash, they should not impact water- or air-borne microfiber pollution. However, this increase in microfiber accumulation on the lint filter by fabric conditioner use only results in a significant decrease in fibers released from the dryer exhaust at the highest dose of fabric conditioner tested. Still, it suggests that promoting fiber accumulation on the lint filter could be used as a potential mechanism to reduce airborne (and subsequent terrestrial and aquatic pollution) arising from dryer exhaust emissions. Although a laundry load comprising 50% polyester T-shirts and 50% cotton T-shirts was used (resulting in a wash load fiber composition of 48% cotton and 52% polyester), analysis of fibers on the dryer lint filter and from the dryer exhaust after the fourth wash cycle (presented in Table 1, full data in S3 Table) conclude that fibers collected are mostly cotton, and fabric conditioner use has no significant impact on the ratio of cotton and polyester fibers collected. The ratio of polyester fibers collected is higher on the lint filter compared to those emitted from the exhaust suggesting that the polyester fibers are more likely to be trapped by the lint filter mesh.

**Fig 4:**
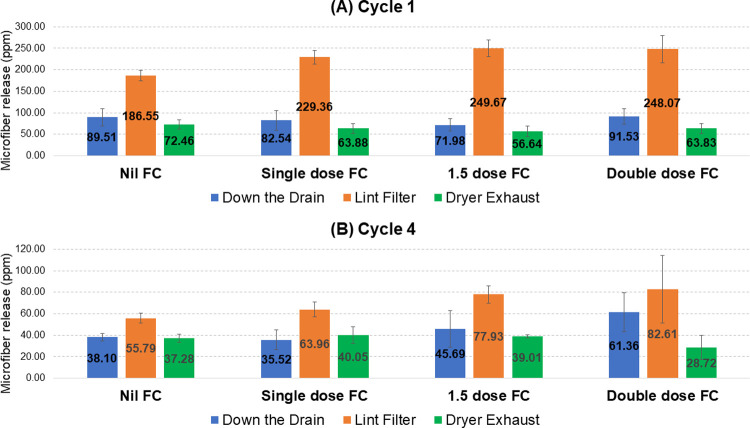
Microfiber release from the first and fourth cycles: North America liquid fabric conditioner testing. Microfiber release during the first (**A**) and fourth (**B**) cycles at each of the three measurement points for all four levels of fabric conditioner (FC) is given in ppm (mg release per kg fabric). Error bars are the standard deviation.

This test suggests that quantities of airborne microfibers being released from the dryer exhaust is only slightly higher than ‘down the drain’ release from the washing machine. However, it is difficult to make a direct comparison of the two from the data in Table 1 as ‘down the drain’ was only measured during the first and fourth cycles. Thus, Fig 4 shows the quantities of microfibers collected ‘down the drain’, at the dryer lint filter and from the dryer exhaust at cycles 1 and 4 (from the full data set in S2 Table). These results show that airborne microfiber release from the tumble dryer exhaust is on average 81.2% of the level measured from ‘down the drain’ release in the wash cycle. This confirms that vented dryer emissions are highly significant, although it is important to recognize that only one appliance type was tested here.

#### Liquid fabric conditioner: European conditions

Similar testing was conducted under European conditions, using detergent (Ariel^®^ pods) and fabric conditioner (Lenor^®^) designed for that region. Results are presented in Table 2, with full data in S4 Table, using the same format as the previous test under North American conditions. The microfiber release data for European conditions shows the same trend as those observed for North America conditions, i.e. fabric conditioner had no significant impact on microfiber release from the washing machine, all three fabric conditioner doses tests caused an increase in dryer lint, and the highest dose tested caused a significant reduction in microfiber release from the tumble dryer exhaust. This provides further evidence that increasing the efficiency of microfiber accumulation on the lint filter could be a useful strategy in reducing airborne emissions from the dryer. The fiber analysis from the exhaust after the fourth cycle (presented in Table 2, full data in S3 Table) also showed the same trend observed under North America conditions of the lint filter being more effective at removing polyester fibers compared to cotton, resulting in a higher ratio of cotton to polyester fibers in the dryer exhaust compared to those collected on the lint filter. In this test, fabric conditioner was also found to significantly increase the ratio of polyester to cotton fibers collected at the lint filter, a trend also observed (although not statistically significant) in the previous test under North America conditions.

**Table 2:** Summary of microfiber release and fiber analysis data–Europe liquid fabric conditioner testing.

	Nil fabric conditioner	Single dose fabric conditioner	1.5 dose fabric conditioner	Double dose fabric conditioner
**Microfiber release**	Average ppm	Average ppm	Average ppm	Average ppm
	Reference	(% change vs Reference)	(% change vs Reference)	(% change vs Reference)
**Down the drain**	79.18	66.63	75.20	84.18
Cycles 1 and 4		(-15.9)	(-5.0)	(+6.3)
**Dryer lint**	107.13	**144.01**	**156.49**	**166.88**
Cycles 1–4		**(+34.4s)**	**(+46.1s)**	**(+55.8s)**
**Dryer exhaust**	48.73	53.04	48.69	**41.81**
Cycles 1–4		(+8.8)	(-0.1)	**(-14.2s)**
**Fiber analysis**	Cotton/Polyester	Cotton/Polyester	Cotton/Polyester	Cotton/Polyester
	%	%	%	%
**Dryer lint**	94.8/5.2	**91.9/8.1s**	**92.0/8.0s**	**93.3/6.7s**
Cycle 4				
**Dryer exhaust**	97.6/2.4	97.3/2.7	96.6/3.4	96.3/3.7
Cycle 4				

**Fig 5:**
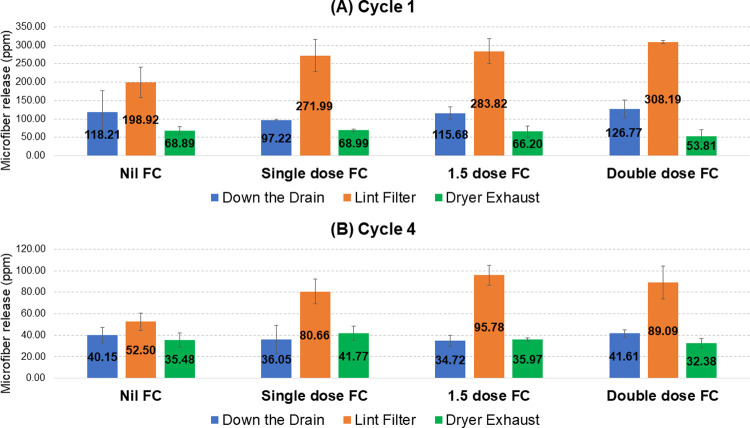
Microfiber release from the first and fourth cycles: Europe liquid fabric conditioner testing. Microfiber release during the first (**A**) and fourth (**B**) cycles at each of the three measurement points for all four levels of fabric conditioner (FC) is given in ppm (mg release per kg fabric). Error bars are the standard deviation.

As with the previous North America test, release data for the first and fourth wash cycles were used to compare relative release at the ‘down the drain’, dryer lint filter and dryer exhaust stages. These data are shown in Fig 5, based on the full data set given in S4 Table. They show that airborne microfiber release from the tumble dryer exhaust is on average 76.8% of the level measured from ‘down the drain’ release in the wash cycle, similar to the 81.2% observed under North America conditions.

#### Liquid anti-wrinkle fabric conditioner

Anti-wrinkle fabric conditioners contain additional functionality to reduce the formation of creases in clothing after washing and drying, resulting in a reduced need for ironing. An example of this product (Downy^®^ WrinkleGuard) was tested under North American conditions using the same methodology as the evaluation of conventional fabric conditioner. Results are shown in Table 3, with full data in S5 Table, showing that all tested doses of anti-wrinkle fabric conditioner have no significant impact on microfiber release from the washing machine and cause a significant increase in microfiber collection on the lint filter of between 43.7–48.5%, in line with observations with conventional fabric conditioner. However, the anti-wrinkle fabric conditioner causes a significant reduction in microfiber release from the dryer vent of between 17.6–35.6% across all tested doses. This will mostly be driven by the anti-wrinkle fabric conditioner increasing efficiency of fiber collection at the lint filter, although other mechanisms might be operating, e.g. the lubricating effect of the anti-wrinkle fabric conditioner could be reducing shedding of microfibers from the garments while tumbling in the dryer. Further studies would be required to fully understand the contribution of different mechanisms to the significant reductions in exhaust microfiber emission. As before, fiber composition from the fourth cycle on the lint filter (presented in Table 3, full data in S3 Table) was richer in polyester than the composition of fibers released from the dryer exhaust although the anti-wrinkle fabric conditioner was found not to significantly impact the ratio of fiber types at either of these points.

**Table 3:** Summary of microfiber release and fiber analysis data–North America liquid anti-wrinkle fabric conditioner testing.

	Nil fabric conditioner	Single dose anti-wrinkle fabric conditioner	1.5 dose anti-wrinkle fabric conditioner	Double dose anti-wrinkle fabric conditioner
**Microfiber release**	Average ppm	Average ppm	Average ppm	Average ppm
	Reference	(% change vs Reference)	(% change vs Reference)	(% change vs Reference)
**Down the drain**	59.44	52.77	53.27	45.42
Cycles 1 and 4		(-11.2)	(-10.4)	(-23.6)
**Dryer lint**	87.26	**125.34**	**129.53**	**128.29**
Cycles 1–4		**(+43.7s)**	**(+48.5s)**	**(+47.0s)**
**Dryer exhaust**	39.29	**32.38**	**29.70**	**25.28**
Cycles 1–4		**(-17.6s)**	**(-24.4s)**	**(-35.6s)**
**Fiber analysis**	Cotton/Polyester	Cotton/Polyester	Cotton/Polyester	Cotton/Polyester
	%	%	%	%
**Dryer lint**	86.4/13.6	85.2/14.8	83.4/16.6	90.0/10.0
Cycle 4				
**Dryer exhaust**	93.0/7.0	93.7/6.3	93.4/6.6	93.2/6.8
Cycle 4				

#### Tumble dryer sheets

**Fig 6:**
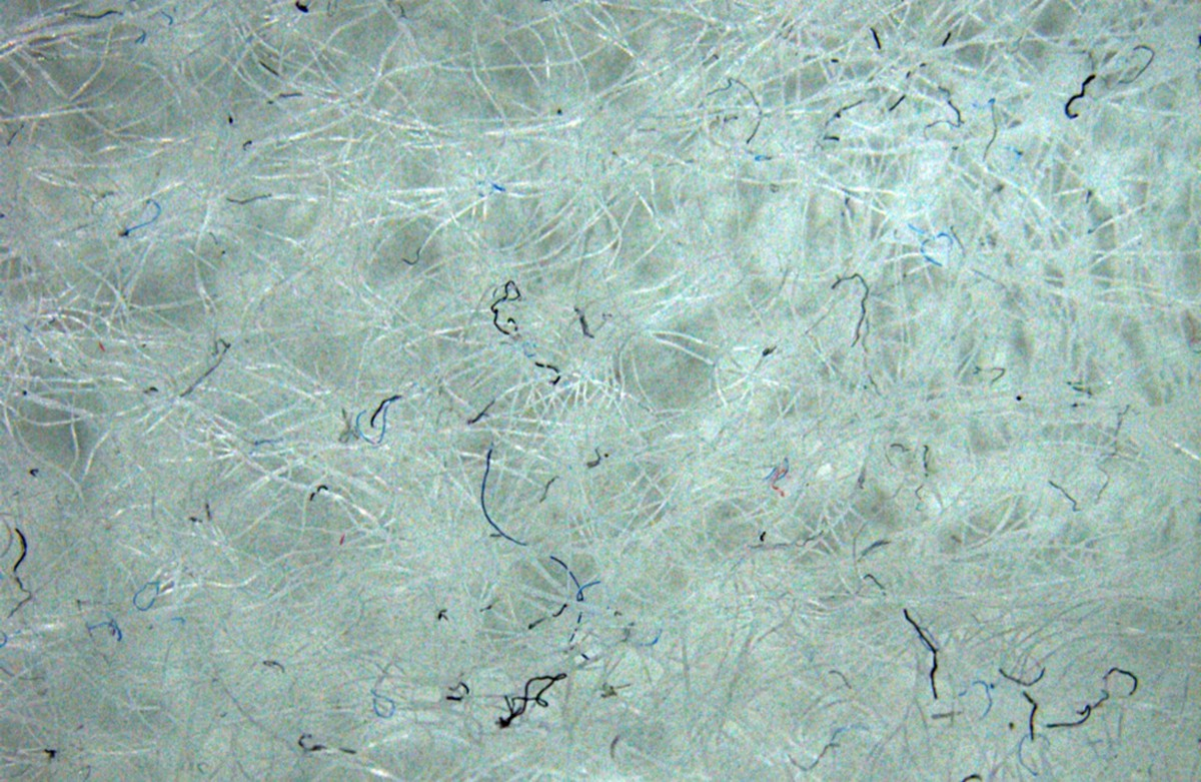
Image of dryer sheet after use.

Tumble dryer sheets are used in some markets to deliver softness, anti-static and freshness benefits to clothing during the drying process. Three usage scenarios were tested involving one or three regular sized sheets (Bounce^®^), or one ‘mega’ sheet (Bounce^®^ WrinkleGuard) using products purchased in North America and tested under North American conditions. As these products are not used in the wash stage, microfiber release was not measured from the washing machine, although fabrics were washed as before using detergent (Tide^®^ pods) prior to each drying cycle. Across all three usage scenarios, dryer sheets were found to significantly reduce microfiber emission from the dryer exhaust by between 14.1–34.9% as shown by the results in Table 4, with full data in S6 Table. However, unlike the testing with liquid fabric conditioners, this was not driven by any increase in microfiber collection on the lint filter. Qualitatively (e.g. Fig 6) the sheets were found to collect microfibers during the drying process and this could be a contributing mechanism to the reduced emission from the dryer exhaust although further studies would be needed to confirm the magnitude of this contribution compared to other mechanisms. The dryer sheets were found not to significantly impact the ratio of released fibers after the fourth cycle although the same trend of fibers collected at the lint filter being richer in polyester content relative to exhaust emissions was observed (Table 4, full data in S3 Table).

**Table 4:** Summary of microfiber release and fiber analysis data–North America dryer sheet testing.

	Nil dryer sheet	1 dryer sheet	3 dryer sheets	1 mega dryer sheet
**Microfiber release**	Average ppm	Average ppm	Average ppm	Average ppm
	Reference	(% change vs Reference)	(% change vs Reference)	(% change vs Reference)
**Dryer lint**	110.96	113.41	105.48	110.11
Cycles 1–4		(+2.2)	(-4.9)	(-0.8)
**Dryer exhaust**	48.47	**41.64**	**31.57**	**33.66**
Cycles 1–4		**(-14.1s)**	**(-34.9s)**	**(-30.6s)**
**Fiber analysis**	Cotton/Polyester	Cotton/Polyester	Cotton/Polyester	Cotton/Polyester
	%	%	%	%
**Dryer lint**	91.4/8.6	91.2/8.8	93.0/7.0	89.8/10.2
Cycle 4				
**Dryer exhaust**	97.2/2.8	95.6/4.4	97.8/2.2	99.8/0.2
Cycle 4				

#### Combination of tumble dryer sheet with liquid anti-wrinkle fabric conditioner

**Fig 7:**
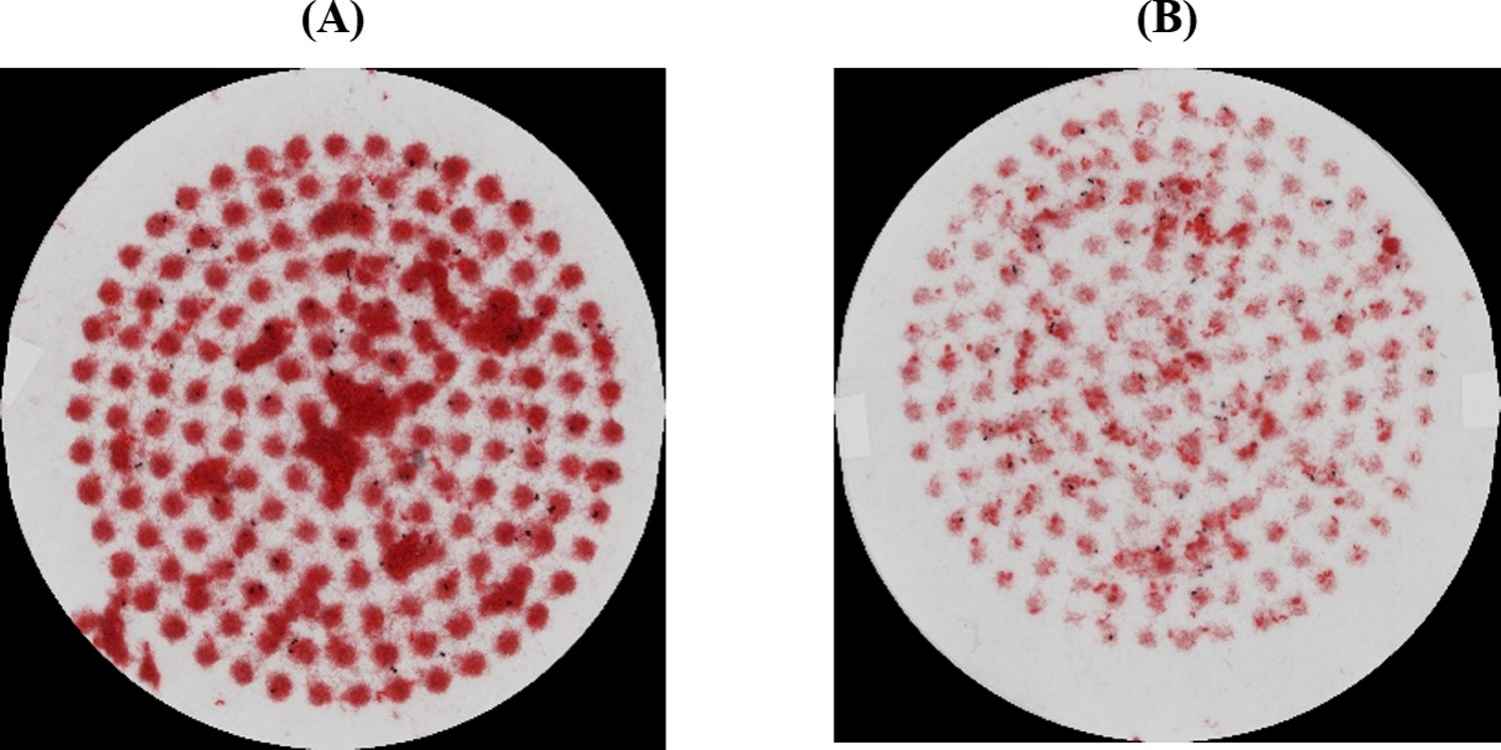
Comparison of filtered fibers collected from the dryer exhaust after the fourth cycle. Paper filter papers from the final Büchner funnel filtration step used to collect fibers from the dryer exhaust of the fourth cycle, comparing the treatment without dryer sheet or anti-wrinkle fabric conditioner (A) with one involving one mega dryer sheet and double dose of anti-wrinkle fabric conditioner (B).

Given that anti-wrinkle liquid fabric conditioner and tumble dryer sheets were both found to significantly reduce emission of microfibers from tumble dryer exhausts, additional testing involving a combined system of both products (double dose of Downy^®^ WrinkleGuard liquid fabric conditioner and use of one Downy^®^ WrinkleGuard mega dryer sheet) was conducted, again using North American products and conditions. While the results (Table 5, full data in S7 Table), showed no significant change in microfibers collected on the dryer lint filter, this combined product system was found to cause a 44.9% reduction in microfiber emission from a dryer exhaust. In line with previous tests, results presented in Table 5 (full data in S3 Table) show that dryer exhaust emissions contained a relatively higher proportion of cotton versus polyester fibers compared to microfibers recovered from the lint filter. While the combined system of anti-wrinkle liquid fabric conditioner and tumble dryer sheets was found to increase the ratio of cotton to polyester fibers collected during the fourth cycle on the lint filter, no significant difference was observed among fibers released from the dryer exhaust. Fig 7 compares the quantity of fibers collected from the dryer exhaust for the two treatments during tumble drying after the fourth wash cycle; in this test red cotton and black polyester T-shirts were used. In line with the fiber composition data, the fiber mass is clearly dominated by cotton.

**Table 5:** Summary of microfiber release and fiber analysis data–North America combination of tumble dryer sheet with liquid anti-wrinkle fabric conditioner.

	Nil dryer sheet or anti-wrinkle fabric conditioner	1 mega dryer sheet + Double dose anti-wrinkle fabric conditioner
**Microfiber release**	Average ppm	Average ppm
	Reference	(% change vs Reference)
**Dryer lint**	99.70	98.93
Cycles 1–4		(-0.8)
**Dryer exhaust**	40.23	**22.18**
Cycles 1–4		**(-44.9s)**
**Fiber analysis**	Cotton/Polyester %	Cotton/Polyester %
**Dryer lint**	90.3/9.7	**96.3/3.7s**
Cycle 4		
**Dryer exhaust**	98.4/1.6	98.6/1.4
Cycle 4		

As the two products combined in this system were both found to significantly reduce microfiber levels from the dryer exhaust when tested alone, it is perhaps unsurprising that their combined use achieves a larger reduction than either of the individual products. However, the incremental benefit appears to confirm that the two products are achieving their reduced microfiber release through the dryer exhaust via different mechanisms.

### Impact of dryer lint filter pore size

Vented tumble dryers contain one or more lint filter devices intended to remove fibers from the warm air prior to release through the exhaust. These filters are designed to be easily removed and cleaned by consumers between drying cycles. The design of the lint filter differs between dryer manufacturers and models and a key point of difference is the pore size of the (typically plastic or metal) mesh used to collect the fibers from the air flow. Kapp and Miller [24] used two tumble dryers in their study, a Roper^®^ model RED4640YQ and LG^®^ model DLEX3570W, reporting a pore size of 1 mm^2^ for both appliances. The Indesit^®^ model IDV75 used in our studies contained a finer pore size of 0.2 mm^2^ which is likely to be significantly more efficient than the dryers used by Kapp and Miller [24] in removing microfibers and hence preventing them from being released through the vent to the outside of the building. Miele^®^ dryers were found to contain finer lint filters with a pore size of 0.04 mm^2^. The impact of pore size on efficiency of microfiber collection was tested by replacing the 0.2 mm^2^ pore size plastic mesh of the Indesit^®^ lint filters used in our study, with 0.04 mm^2^ pore size mesh (as removed from Miele^®^) lint filters and carrying out tests under European conditions with detergent (1 Ariel^®^ pod per wash) and no fabric conditioner. Results, shown in Table 6, with full data in S8 Table, confirm that moving from a 0.2 mm^2^ to 0.04 mm^2^ pore size mesh delivers a significant 34.8% reduction in microfiber release through the dryer exhaust. In line with the other tests, fibers collected on the lint filter during the fourth cycle contained a relatively higher proportion of polyester fibers compared to those emitted through the exhaust, although the change in lint filter pore size did not significantly impact these ratios (Table 6, full data in S3 Table). The present study involved lint filters with pore sizes of 0.2 mm^2^ and 0.04 mm^2^ yet work reported by Kapp and Miller [24] and our own cursory inspection of various vented tumble dryers sold on the U.S.A. market suggests that many involve pore sizes of around 1mm^2^. This suggests that airborne microfiber release levels from dryer exhausts in North America could be even higher than ‘down the drain’ release from the wash cycle given that our North America testing with a 0.2 mm^2^ lint filter showed only slightly lower (81.2% of the total) levels of airborne release from the dryer exhaust compared to ‘down the drain’.

**Table 6:** Summary of microfiber release and fiber analysis data–impact of lint filter pore size.

	Coarse (0.2 mm^2^) pore size	Fine (0.04 mm^2^) pore size
	lint filter	lint filter
**Microfiber release**	Average ppm	Average ppm
	Reference	(% change vs Reference)
**Dryer lint**	99.29	120.81
Cycles 1–4		(+21.7)
**Dryer exhaust**	48.27	**31.48**
Cycles 1–4		**(-34.8s)**
**Fiber analysis**	Cotton/Polyester %	Cotton/Polyester %
**Dryer lint**	93.0/7.0	93.0/7.0
Cycle 4		
**Dryer exhaust**	98.3/1.7	96.0/4.0
Cycle 4		

### Sheddability testing and fiber dimensions

Tape lift measurements were conducted to measure the intrinsic sheddability of the two test garments. Results are given in Table 7 (full data in S9 Table), showing that the cotton T-shirt releases over twenty times the number of fibers versus the polyester T-shirt. This is in line with the significant excess of cotton fibers collected on the lint filter and released from the dryer exhaust, raising a possibility that tape lift experiments could form the basis of a screening method used to compare the sheddability of different textiles or garments. The recent applications of tape lifts for microplastic quantification on filter papers [30] and contactless airborne transfer of fibers during the wearing of clothes [19] further supports the value of such methods in microfiber research.

**Table 7:** Summary of tape lift sheddability data for the tested garments.

Number of fibers lifted within each 1 cm^2^ window (n = 6), mean ± SD	
100% Cotton T-Shirt	100% Polyester T-Shirt
17.83 **±** 9.20	0.83 **±** 0.75

A sample of fibers collected by tape lift from each of the two garments was analyzed to determine average length and width, with results given in Table 8 (full data in S10 Table). These show the expected fiber widths for cotton and polyester microfiber of 23.1 μm and 12.6 μm, respectively. The lengths of fiber fragments determined were 3.8 mm and 1.4 mm for cotton and polyester, respectively. The distribution of lengths observed suggest that tape lifting is removing small fragments of fibers rather than (in the case of cotton) full staple fibers which are typically >9 mm in length. Further work would be needed to determine whether the fiber size distribution collected by tape lifting is similar to that released during the washing or drying processes.

**Table 8:** Average fiber length and width of fibers collected by tape lift.

	Fiber dimensions (n = 20), mean ± SD	
	100% Cotton T-Shirt	100% Polyester T-Shirt
**Length (mm)**	3.78 **±** 2.11	1.40 **±** 2.28
**Width (μm)**	23.06 **±** 6.03	12.62 **±** 1.64

## Conclusions

Tumble drying generates significant quantities of microfibers, far more than the quantities released to the drain during the textile washing step. The lint filter can capture many of the fibers released during tumble drying and hence prevent airborne pollution, but the efficiency of this process will depend on the design of the filters including its pore size. As cotton garments typically release more fibers than those constructed from polyester, and as lint filters appear to be relatively more effective in trapping polyester over cotton fibers, most microfiber pollution arising from dryers is likely to involve cotton fibers which tend to be more biodegradable than fibers of synthetic origin.

While more work is needed to fully understand the environmental impact of microfiber pollution from vented tumble dryers, this study confirms that fabric care products designed to condition textiles may help mitigate the issue as each product tested led to a significant reduction in microfiber release from the dryer, although high doses of regular liquid fabric conditioner were needed to have a significant benefit. The study also provides important insights for the appliance industry which is currently manufacturing dryers with a broad range of lint filters with pore sizes of between ≤0.020 mm^2^ up to ≥1 mm^2^. There is a clear opportunity for these manufacturers to reduce the quantity of microfiber emissions from vented dryer exhausts by improving the performance of lint removal systems. This could be achieved by optimizing lint filter pore size or moving to a cyclonic separation system.

Legislators will need to consider whether current airborne microfiber release levels from vented tumble dryers are acceptable or require interventions such as improved appliance design or driving conversion to fully-sealed condenser dryers. Further studies are recommended to compare airborne release from clothing during line drying with tumble drying and understand whether textile construction improvements known to reduce ‘down the drain’ microfiber release also deliver reduced airborne release from vented dryer vents. More consumer-relevant tests involving real soiled mixed laundry loads would also provide useful insights to guide policy-making and improved appliance design.

Condenser boxes are commercially available to enable internal venting of tumble dryers by removing water from the exhaust prior to discharge into the room. It is possible that these devices do not efficiently remove discharged microfibers raising potential human health issues arising from their inhalation. It is recommended that the manufacturers and suppliers of condenser boxes confirm that they do not raise such health issues when used in accordance with their operating instructions.

Finally, the present studies all involved collection of microfibers released ‘down the drain’ or through dryer exhausts using 20 μm CellMicroSieve^®^ filters. Smaller particle size microplastic debris could have been released from the clothing which passed through these filters, raising further risks to human and environmental safety. More studies are needed to understand the relevance of this <20 μm aspect of the issue.

## Supporting information

S1 Table Details of garments tested. All test loads comprised ten 100% cotton T-shirts (Fruit of the Loom^®^ Original T-shirt, product code 61–082, size L) and ten 100% polyester T-shirts (Fruit of the Loom^®^ Performance T-shirt, product code 61–390, size L). The table shows the garment colors and wholesale supplier used for T-shirts in each test.
(DOCX)

S2 Table Microfiber release data–North America liquid fabric conditioner testing. The table shows measured mass of the wash load used (kg) and microfibers collected (mg) for microfiber release ‘down the drain’ (cycles 1 and 4), collected on the dryer lint filter (cycles 1–4) and released from dryer exhaust (cycles 1–4). These data are used to calculate quantity of microfibers at these three stages in terms of ppm (parts per million, i.e. mg microfiber released per kg dry wash load).
(DOCX)

S3 Table Fiber composition analysis data. (DOCX)

S4 Table Microfiber release data–Europe liquid fabric conditioner testing. The table shows measured mass of the wash load used (kg) and microfibers collected (mg) for microfiber release ‘down the drain’ (cycles 1 and 4), collected on the dryer lint filter (cycles 1–4) and released from dryer exhaust (cycles 1–4). These data are used to calculate quantity of microfibers at these three stages in terms of ppm (parts per million, i.e. mg microfiber released per kg dry wash load).
(DOCX)

S5 Table Microfiber release data–North America liquid anti-wrinkle fabric conditioner testing. The table shows measured mass of the wash load used (kg) and microfibers collected (mg) for microfiber release ‘down the drain’ (cycles 1 and 4), collected on the dryer lint filter (cycles 1–4) and released from dryer exhaust (cycles 1–4). These data are used to calculate quantity of microfibers at these three stages in terms of ppm (parts per million, i.e. mg microfiber released per kg dry wash load).
(DOCX)

S6 Table Microfiber release data–North America dryer sheet testing. The table shows measured mass of the wash load used (kg) and microfibers collected (mg) for microfibers collected on the dryer lint filter (cycles 1–4) and released from dryer exhaust (cycles 1–4). These data are used to calculate quantity of microfibers at both of these stages in terms of ppm (parts per million, i.e. mg microfiber released per kg dry wash load).
(DOCX)

S7 Table Microfiber release data–North America combination of tumble dryer. Sheet with liquid anti-wrinkle fabric conditioner. The table shows measured mass of the wash load used (kg) and microfibers collected (mg) for microfibers collected on the dryer lint filter (cycles 1–4) and released from dryer exhaust (cycles 1–4). These data are used to calculate quantity of microfibers at both of these stages in terms of ppm (parts per million, i.e. mg microfiber released per kg dry wash load).
(DOCX)

S8 Table Microfiber release data–impact of lint filter pore size. The table shows measured mass of the wash load used (kg) and microfibers collected (mg) for microfibers collected on the dryer lint filter (cycles 1–4) and released from dryer exhaust (cycles 1–4). These data are used to calculate quantity of microfibers at both of these stages in terms of ppm (parts per million, i.e. mg microfiber released per kg dry wash load).
(DOCX)

S9 Table Tape lift sheddability data for the tested garments. The table shows the number of fibers counted within 1 cm^2^ windows tape-lifted from the 100% Cotton and 100% Polyester T-shirts used in the testing.
(DOCX)

S10 Table Fiber length and width data for fibers collected by tape lift. The table shows the fiber length and width of fibers collected by tape lifting from the 100% Cotton and 100% Polyester T-shirts used in the testing.
(DOCX)
