# Supplementary material for: The impact of fabric conditioning products and lint filter pore size on airborne microfiber pollution arising from tumble drying
Source: PLoS One. 2022 Apr 6;17(4):e0265912. doi: 10.1371/journal.pone.0265912 (PMC8985936; doi:10.1371/journal.pone.0265912)
Supplement: S1 Table — All test loads comprised ten 100% cotton T-shirts (Fruit of the Loom® Original T-shirt, product code 61–082, size L) and ten 100% polyester T-shirts (Fruit of the Loom® Performance T-shirt, product code 61–390, size L). The table shows the garment colors and wholesale supplier used for T-shirts in each test. (DOCX) [file pone.0265912.s001.docx]

**S1** **Table. Details of garments tested.** All test loads comprised ten 100% cotton T-shirts (Fruit of the Loom^®^ Original T-shirt, product code 61-082, size L) and ten 100% polyester T-shirts (Fruit of the Loom^®^ Performance T-shirt, product code 61-390, size L). The table shows the garment colors and wholesale supplier used for T-shirts in each test.

| **Test** | **T-shirt color** | **Supplier** |
| --- | --- | --- |
| **Liquid fabric conditioner: North America conditions**  (results summarized in Table 1) | Cotton: Red  Polyester: Black | BTC* |
| **Liquid fabric conditioner: European conditions**  (results summarized in Table 2) | Cotton: Red  Polyester: Black | BTC* |
| **Liquid anti-wrinkle fabric conditioner**  (results summarized in Table 3) | Cotton: Sky blue  Polyester: Black | Cotton: BTC*  Polyester: MDP** |
| **Tumble dryer sheets**  (results summarized in Table 4) | Cotton: Black  Polyester: Royal blue | BTC* |
| **Combination of tumble dryer sheet with liquid anti-wrinkle fabric conditioner**  (results summarized in Table 5) | Cotton: Red  Polyester: Black/ Dark blue | BTC* |
| **Impact of dryer lint filter pore size**  (results summarized in Table 6) | Cotton: Red  Polyester: Black | BTC* |
| **Sheddability testing and fiber dimensions** | Cotton: Red  Polyester: Black | BTC* |

***** BTC Activewear Limited., Wednesbury, U.K

** MDP supplies, Bromborough, UK.
