# Supplementary material for: The impact of fabric conditioning products and lint filter pore size on airborne microfiber pollution arising from tumble drying
Source: PLoS One. 2022 Apr 6;17(4):e0265912. doi: 10.1371/journal.pone.0265912 (PMC8985936; doi:10.1371/journal.pone.0265912)
Supplement: S2 Table — The table shows measured mass of the wash load used (kg) and microfibers collected (mg) for microfiber release ‘down the drain’ (cycles 1 and 4), collected on the dryer lint filter (cycles 1–4) and released from dryer exhaust (cycles 1–4). These data are used to calculate quantity of microfibers at these three stages in terms of ppm (parts per million, i.e. mg microfiber released per kg dry wash load). (DOCX) [file pone.0265912.s002.docx]

**S2** **Table. Microfiber release data – North America liquid fabric conditioner testing.** The table shows measured mass of the wash load used (kg) and microfibers collected (mg) for microfiber release down the drain (cycles 1 and 4), collected on the dryer lint filter (cycles 1-4) and released from dryer exhaust (cycles 1-4). These data are used to calculate quantity of microfibers at these three stages in terms of ppm (parts per million, i.e. mg microfiber released per kg dry wash load).

| **Nil fabric conditioner** | | | |  |  |  |  |
| --- | --- | --- | --- | --- | --- | --- | --- |
|  | **Load mass**  **(kg)** | **Microfiber mass**  **(mg)** | | | **Microfiber release**  **(ppm)*** | | |
|  |  | Down the drain | Lint filter | Dryer Exhaust | Down the drain | Lint filter | Dryer Exhaust |
| Cycle 1 – Load 1 | 2.970 | 298.90 | 577.50 | 178.30 | 100.64 | 194.44 | 60.03 |
| Cycle 1 – Load 2 | 3.010 | 199.70 | 519.00 | 234.60 | 66.35 | 172.43 | 77.94 |
| Cycle 1 – Load 3 | 2.960 | 300.50 | 570.60 | 235.00 | 101.52 | 192.77 | 79.39 |
| **Cycle 1 - Mean** | **2.980** | **266.37** | **555.70** | **215.97** | **89.50** | **186.55** | **72.46** |
| **Cycle 1 – Std Dev** | **0.026** | **57.74** | **31.97** | **32.62** | **20.06** | **12.26** | **10.78** |
| Cycle 2 – Load 1 | 2.970 | - | 314.90 | 181.10 | - | 106.03 | 60.98 |
| Cycle 2 – Load 2 | 3.010 | - | 392.40 | 154.90 | - | 130.37 | 51.46 |
| Cycle 2 – Load 3 | 2.960 | - | 274.30 | 180.40 | - | 92.67 | 60.95 |
| **Cycle 2 - Mean** | **2.980** | - | **327.20** | **172.13** | - | **109.69** | **57.79** |
| **Cycle 2 – Std Dev** | **0.026** | **-** | **60.00** | **14.93** | **-** | **19.11** | **5.48** |
| Cycle 3 – Load 1 | 2.970 | - | 190.00 | 90.10 | - | 63.97 | 30.34 |
| Cycle 3 – Load 2 | 3.010 | - | 246.40 | 88.80 | - | 81.86 | 29.50 |
| Cycle 3 – Load 3 | 2.960 | - | 209.00 | 120.00 | - | 70.61 | 40.54 |
| **Cycle 3 - Mean** | **2.980** | - | **215.13** | **99.63** | - | **72.15** | **33.46** |
| **Cycle 3 – Std Dev** | **0.026** | **-** | **28.70** | **17.65** | **-** | **9.04** | **6.15** |
| Cycle 4 – Load 1 | 2.970 | 122.60 | 176.50 | 116.30 | 41.28 | 59.43 | 39.16 |
| Cycle 4 – Load 2 | 3.010 | 115.50 | 173.00 | 99.10 | 38.37 | 57.48 | 32.92 |
| Cycle 4 – Load 3 | 2.960 | 102.60 | 149.30 | 117.70 | 34.66 | 50.44 | 39.76 |
| **Cycle 4 - Mean** | **2.980** | **113.57** | **166.27** | **111.03** | **38.10** | **55.78** | **37.28** |
| **Cycle 4 – Std Dev** | **0.026** | **10.14** | **14.80** | **10.36** | **3.32** | **4.73** | **3.79** |
| **Single dose fabric conditioner** | | | | | | | |
|  | **Load mass**  **(kg)** | **Microfiber mass**  **(mg)** | | | **Microfiber release**  **(ppm)*** | | |
|  |  | Down the drain | Lint filter | Dryer Exhaust | Down the drain | Lint filter | Dryer Exhaust |
| Cycle 1 – Load 1 | 2.970 | 242.00 | 715.40 | 153.60 | 81.48 | 240.88 | 51.72 |
| Cycle 1 – Load 2 | 3.030 | 182.50 | 714.60 | 198.00 | 60.23 | 235.84 | 65.35 |
| Cycle 1 – Load 3 | 2.970 | 314.50 | 627.80 | 221.50 | 105.89 | 211.38 | 74.58 |
| **Cycle 1 - Mean** | **2.990** | **246.33** | **685.93** | **191.03** | **82.53** | **229.37** | **63.88** |
| **Cycle 1 – Std Dev** | **0.035** | **66.11** | **50.35** | **34.48** | **22.85** | **15.78** | **11.50** |
| Cycle 2 – Load 1 | 2.970 | - | 371.20 | 171.90 | - | 124.98 | 57.88 |
| Cycle 2 – Load 2 | 3.030 | - | 469.90 | 216.90 | - | 155.08 | 71.58 |
| Cycle 2 – Load 3 | 2.970 | - | 390.30 | 191.90 | - | 131.41 | 64.61 |
| **Cycle 2 - Mean** | **2.990** | - | **410.47** | **193.57** | - | **137.16** | **64.69** |
| **Cycle 2 – Std Dev** | **0.035** | **-** | **52.35** | **22.55** | **-** | **15.85** | **6.85** |
| Cycle 3 – Load 1 | 2.970 | - | 237.80 | 92.90 | - | 80.07 | 31.28 |
| Cycle 3 – Load 2 | 3.030 | - | 219.30 | 139.70 | - | 72.38 | 46.11 |
| Cycle 3 – Load 3 | 2.970 | - | 246.20 | 110.50 | - | 82.90 | 37.21 |
| **Cycle 3 - Mean** | **2.990** | - | **234.43** | **114.37** | - | **78.45** | **38.20** |
| **Cycle 3 – Std Dev** | **0.035** | **-** | **13.76** | **23.64** | **-** | **5.44** | **7.46** |
| Cycle 4 – Load 1 | 2.970 | 128.90 | 201.90 | 144.80 | 43.40 | 67.98 | 48.75 |
| Cycle 4 – Load 2 | 3.030 | 114.40 | 168.90 | 101.20 | 37.76 | 55.74 | 33.40 |
| Cycle 4 – Load 3 | 2.970 | 75.40 | 202.50 | 112.80 | 25.39 | 68.18 | 37.98 |
| **Cycle 4 - Mean** | **2.990** | **106.23** | **191.10** | **119.60** | **35.51** | **63.97** | **40.04** |
| **Cycle 4 – Std Dev** | **0.035** | **27.67** | **19.23** | **22.58** | **9.21** | **7.12** | **7.88** |
| **1.5 dose fabric conditioner** | | | | | | | |
|  | **Load mass**  **(kg)** | **Microfiber mass**  **(mg)** | | | **Microfiber release**  **(ppm)*** | | |
|  |  | Down the drain | Lint filter | Dryer Exhaust | Down the drain | Lint filter | Dryer Exhaust |
| Cycle 1 – Load 1 | 2.970 | 261.3 | 737.7 | 199.5 | 87.98 | 248.38 | 67.17 |
| Cycle 1 – Load 2 | 3.030 | 180.5 | 700.6 | 181.4 | 59.57 | 231.22 | 59.87 |
| Cycle 1 – Load 3 | 2.99 | 204.5 | 805.5 | 128.2 | 68.39 | 269.40 | 42.88 |
| **Cycle 1 - Mean** | **2.997** | **215.43** | **747.93** | **169.70** | **71.98** | **249.67** | **56.64** |
| **Cycle 1 – Std Dev** | **0.030** | **41.49** | **53.19** | **37.06** | **14.54** | **19.12** | **12.47** |
| Cycle 2 – Load 1 | 2.970 | - | 414.2 | 140 | - | 139.46 | 47.14 |
| Cycle 2 – Load 2 | 3.030 | - | 440.1 | 139.2 | - | 145.25 | 45.94 |
| Cycle 2 – Load 3 | 2.990 | - | 377.9 | 151 | - | 126.39 | 50.50 |
| **Cycle 2 - Mean** | **2.997** | - | **410.73** | **143.40** | - | **137.03** | **47.86** |
| **Cycle 2 – Std Dev** | **0.030** | **-** | **31.24** | **6.59** | **-** | **9.66** | **2.36** |
| Cycle 3 – Load 1 | 2.970 | - | 253.2 | 83.2 | - | 85.25 | 28.01 |
| Cycle 3 – Load 2 | 3.030 | - | 276.8 | 105.4 | - | 91.35 | 34.79 |
| Cycle 3 – Load 3 | 2.990 | - | 271.7 | 107.9 | - | 90.87 | 36.09 |
| **Cycle 3 - Mean** | **2.997** | - | **267.23** | **98.83** | - | **89.16** | **32.96** |
| **Cycle 3 – Std Dev** | **0.030** | **-** | **12.42** | **13.60** | **-** | **3.39** | **4.33** |
| Cycle 4 – Load 1 | 2.970 | 190.6 | 258.3 | 115.8 | 64.18 | 86.97 | 38.99 |
| Cycle 4 – Load 2 | 3.030 | 92.6 | 225.0 | 122.9 | 30.56 | 74.26 | 40.56 |
| Cycle 4 – Load 3 | 2.990 | 126.6 | 217.0 | 112.1 | 42.34 | 72.58 | 37.48 |
| **Cycle 4 - Mean** | **2.997** | **136.6** | **233.43** | **116.93** | **45.69** | **77.93** | **39.01** |
| **Cycle 4 – Std Dev** | **0.030** | **69.30** | **23.55** | **5.02** | **17.06** | **7.87** | **1.54** |
| **Double dose fabric conditioner** | | | | | | | |
|  | **Load mass**  **(kg)** | **Microfiber mass**  **(mg)** | | | **Microfiber release**  **(ppm)*** | | |
|  |  | Down the drain | Lint filter | Dryer Exhaust | Down the drain | Lint filter | Dryer Exhaust |
| Cycle 1 – Load 1 | 2.980 | 255.10 | 698.40 | 194.00 | 85.60 | 234.36 | 65.10 |
| Cycle 1 – Load 2 | 3.030 | 233.80 | 684.20 | 225.60 | 77.16 | 225.81 | 74.46 |
| Cycle 1 – Load 3 | 2.990 | 334.30 | 849.30 | 155.30 | 111.81 | 284.05 | 51.94 |
| **Cycle 1 - Mean** | **3.000** | **274.40** | **743.97** | **191.63** | **91.52** | **248.07** | **63.83** |
| **Cycle 1 – Std Dev** | **0.027** | **52.96** | **91.50** | **35.21** | **18.06** | **31.45** | **11.31** |
| Cycle 2 – Load 1 | 2.980 | - | 377.40 | 116.30 | - | 126.64 | 39.03 |
| Cycle 2 – Load 2 | 3.030 | - | 372.20 | 123.70 | - | 122.84 | 40.83 |
| Cycle 2 – Load 3 | 2.990 | - | 377.20 | 155.30 | - | 126.15 | 51.94 |
| **Cycle 2 - Mean** | **3.000** | - | **375.60** | **131.77** | - | **125.21** | **43.93** |
| **Cycle 2 – Std Dev** | **0.027** | **-** | **2.95** | **20.71** | **-** | **2.07** | **6.99** |
| Cycle 3 – Load 1 | 2.980 | - | 237.70 | 62.10 | - | 79.77 | 20.84 |
| Cycle 3 – Load 2 | 3.030 | - | 339.10 | 62.80 | - | 111.91 | 20.73 |
| Cycle 3 – Load 3 | 2.990 | - | 279.80 | 64.50 | - | 93.58 | 21.57 |
| **Cycle 3 - Mean** | **3.000** | - | **285.53** | **63.13** | - | **95.09** | **21.05** |
| **Cycle 3 – Std Dev** | **0.027** | **-** | **50.94** | **1.23** | **-** | **16.13** | **0.46** |
| Cycle 4 – Load 1 | 2.980 | 175.70 | 268.90 | 79.30 | 58.96 | 90.23 | 26.61 |
| Cycle 4 – Load 2 | 3.030 | 220.80 | 225.30 | 107.20 | 72.87 | 74.36 | 35.38 |
| Cycle 4 – Load 3 | 2.990 | 156.30 | 248.90 | 72.20 | 52.27 | 83.24 | 24.15 |
| **Cycle 4 - Mean** | **3.000** | **184.27** | **247.70** | **86.23** | **61.37** | **82.61** | **28.71** |
| **Cycle 4 – Std Dev** | **0.027** | **33.09** | **21.82** | **18.50** | **10.51** | **7.96** | **5.90** |

***Microfiber release (ppm) = Microfiber mass (mg) / Load mass (kg)**
