# Supplementary material for: The impact of fabric conditioning products and lint filter pore size on airborne microfiber pollution arising from tumble drying
Source: PLoS One. 2022 Apr 6;17(4):e0265912. doi: 10.1371/journal.pone.0265912 (PMC8985936; doi:10.1371/journal.pone.0265912)
Supplement: S3 Table — (DOCX) [file pone.0265912.s003.docx]

**S3** **Table. Fiber composition analysis data.** The table shows the analyzed fiber composition of the microfibers collected on the lint filter and from the dryer exhaust for all testing.

| **North America liquid fabric conditioner testing** | | | | | | | |
| --- | --- | --- | --- | --- | --- | --- | --- |
|  | **Lint filter** | | | | | **Dryer Exhaust** | |
| **Nil fabric conditioner** | % Polyester | | | % Cotton | | % Polyester | % Cotton |
| Load 1 | 7.7 | | | 92.3 | | 3.9 | 96.1 |
| Load 2 | 9.8 | | | 90.2 | | 4.5 | 95.5 |
| Load 3 | 8.6 | | | 91.4 | | 2.8 | 97.2 |
| **Average** | **8.7** | | | **91.3** | | **3.7** | **96.3** |
| Standard deviation | 1.0 | | | 1.0 | | 0.8 | 0.8 |
| **Single dose fabric conditioner** | % Polyester | | | % Cotton | | % Polyester | % Cotton |
| Load 1 | 9.6 | | | 90.4 | | 3.5 | 96.5 |
| Load 2 | 10.8 | | | 89.2 | | 6.1 | 93.9 |
| Load 3 | 13.6 | | | 86.4 | | 1.0 | 99.0 |
| **Average** | **11.3** | | | **88.7** | | **3.5** | **96.5** |
| Standard deviation | 2.1 | | | 2.1 | | 2.6 | 2.6 |
| **1.5 dose fabric conditioner** | % Polyester | | | % Cotton | | % Polyester | % Cotton |
| Load 1 | 14.2 | | | 85.8 | | 5.5 | 94.5 |
| Load 2 | 9.1 | | | 90.9 | | 4.6 | 95.4 |
| Load 3 | 14.2 | | | 85.8 | | 3.3 | 96.7 |
| **Average** | **12.5** | | | **87.5** | | **4.5** | **95.5** |
| Standard deviation | 2.9 | | | 2.9 | | 1.1 | 1.1 |
| **Double dose fabric conditioner** | % Polyester | | | % Cotton | | % Polyester | % Cotton |
| Load 1 | 8.7 | | | 91.3 | | 1.7 | 98.3 |
| Load 2 | 6.3 | | | 93.7 | | 2.9 | 97.1 |
| Load 3 | 8.1 | | | 91.9 | | 1.7 | 98.3 |
| **Average** | **7.7** | | | **92.3** | | **2.1** | **97.9** |
| Standard deviation | 1.3 | | | 1.3 | | 0.7 | 0.7 |
| **Europe liquid fabric conditioner testing** | | | | | | | |
|  | **Lint filter** | | | | | **Dryer Exhaust** | |
| **Nil fabric conditioner** | % Polyester | | | % Cotton | | % Polyester | % Cotton |
| Load 1 | 5.4 | | | 94.6 | | 1.9 | 98.1 |
| Load 2 | 5.7 | | | 94.3 | | 1.9 | 98.1 |
| Load 3 | 4.5 | | | 95.5 | | 3.4 | 96.6 |
| **Average** | **5.2** | | | **94.8** | | **2.4** | **97.6** |
| Standard deviation | 0.6 | | | 0.6 | | 0.9 | 0.9 |
| **Single dose fabric conditioner** | % Polyester | | | % Cotton | | % Polyester | % Cotton |
| Load 1 | 8.8 | | | 91.2 | | 5.5 | 94.5 |
| Load 2 | 9.0 | | | 91.0 | | 0.4 | 99.6 |
| Load 3 | 6.3 | | | 93.7 | | 2.2 | 97.8 |
| **Average** | **8.1** | | | **91.9** | | **2.7** | **97.3** |
| Standard deviation | 1.5 | | | 1.5 | | 2.6 | 2.6 |
| **1.5 dose fabric conditioner** | % Polyester | | | % Cotton | | % Polyester | % Cotton |
| Load 1 | 8.8 | | | 91.2 | | 1.6 | 98.4 |
| Load 2 | 7.6 | | | 92.4 | | 5.6 | 94.4 |
| Load 3 | 7.7 | | | 92.3 | | 3.0 | 97.0 |
| **Average** | **8.0** | | | **92.0** | | **3.4** | **96.6** |
| Standard deviation | 0.7 | | | 0.7 | | 2.0 | 2.0 |
| **Double dose fabric conditioner** | % Polyester | | | % Cotton | | % Polyester | % Cotton |
| Load 1 | 6.2 | | | 93.8 | | 5.3 | 94.7 |
| Load 2 | 7.2 | | | 92.8 | | 4.2 | 95.8 |
| Load 3 | 6.8 | | | 93.2 | | 1.7 | 98.3 |
| **Average** | **6.7** | | | **93.3** | | **3.7** | **96.3** |
| Standard deviation | 0.5 | | | 0.5 | | 1.8 | 1.8 |
| **North America liquid anti-wrinkle fabric conditioner testing** | | | | | | | |
|  | | **Lint filter** | | | **Dryer Exhaust** | | |
| **Nil fabric conditioner** | | % Polyester | % Cotton | | % Polyester | | % Cotton |
| Load 1 | | 9.8 | 90.2 | | 8.3 | | 91.7 |
| Load 2 | | 11.0 | 89.0 | | 5.8 | | 94.2 |
| Load 3 | | 20.0 | 80.0 | | 7.0 | | 93.0 |
| **Average** | | **13.6** | **86.4** | | **7.0** | | **93.0** |
| Standard deviation | | 5.6 | 5.6 | | 1.3 | | 1.3 |
| **Single dose anti-wrinkle fabric conditioner** | | % Polyester | % Cotton | | % Polyester | | % Cotton |
| Load 1 | | 14.8 | 85.2 | | 7.9 | | 92.1 |
| Load 2 | | 20.3 | 79.7 | | 4.9 | | 95.1 |
| Load 3 | | 9.3 | 90.7 | | 6.1 | | 93.9 |
| **Average** | | **14.8** | **85.2** | | **6.3** | | **93.7** |
| Standard deviation | | 5.5 | 5.5 | | 1.5 | | 1.5 |
| **1.5 dose anti-wrinkle**  **fabric conditioner** | | % Polyester | % Cotton | | % Polyester | | % Cotton |
| Load 1 | | 29.5 | 70.5 | | 6.6 | | 93.4 |
| Load 2 | | 9.3 | 90.7 | | 4.7 | | 95.3 |
| Load 3 | | 11.0 | 89.0 | | 8.4 | | 91.6 |
| **Average** | | **16.6** | **83.4** | | **6.6** | | **93.4** |
| Standard deviation | | 11.2 | 11.2 | | 1.9 | | 1.9 |
| **Double dose anti-wrinkle fabric conditioner** | | % Polyester | % Cotton | | % Polyester | | % Cotton |
| Load 1 | | 10.3 | 89.7 | | 7.8 | | 92.2 |
| Load 2 | | 8.9 | 91.1 | | 7.1 | | 92.9 |
| Load 3 | | 10.8 | 89.2 | | 5.4 | | 94.6 |
| **Average** | | **10.0** | **90.0** | | **6.8** | | **93.2** |
| Standard deviation | | 1.0 | 1.0 | | 1.3 | | 1.3 |
| **North America dryer sheet testing** | | | | | | | |
|  | | **Lint filter** | | | **Dryer Exhaust** | | |
| **Nil dryer sheet** | | % Polyester | % Cotton | | % Polyester | | % Cotton |
| Load 1 | | 7.7 | 92.3 | | 3.9 | | 96.1 |
| Load 2 | | 10.0 | 90.0 | | 0.7 | | 99.3 |
| Load 3 | | 8.2 | 91.8 | | 3.9 | | 96.1 |
| **Average** | | **8.6** | **91.4** | | **2.8** | | **97.2** |
| Standard deviation | | 1.2 | 1.2 | | 1.9 | | 1.9 |
| **1 dryer sheet** | | % Polyester | % Cotton | | % Polyester | | % Cotton |
| Load 1 | | 8.7 | 91.3 | | 5.8 | | 94.2 |
| Load 2 | | 7.5 | 92.5 | | 2.3 | | 97.7 |
| Load 3 | | 10.1 | 89.9 | | 5.1 | | 94.9 |
| **Average** | | **8.8** | **91.2** | | **4.4** | | **95.6** |
| Standard deviation | | 1.3 | 1.3 | | 1.9 | | 1.9 |
| **3 dryer sheets** | | % Polyester | % Cotton | | % Polyester | | % Cotton |
| Load 1 | | 4.6 | 95.4 | | 2.2 | | 97.8 |
| Load 2 | | 6.1 | 93.9 | | 3.2 | | 96.8 |
| Load 3 | | 10.3 | 89.7 | | 1.2 | | 98.8 |
| **Average** | | **7.0** | **93.0** | | **2.2** | | **97.8** |
| Standard deviation | | 2.9 | 2.9 | | 1.0 | | 1.0 |
| **1 mega dryer sheet** | | % Polyester | % Cotton | | % Polyester | | % Cotton |
| Load 1 | | 8.4 | 91.6 | | 0.4 | | 99.6 |
| Load 2 | | 9.7 | 90.3 | | 0.0 | | 100.0 |
| Load 3 | | 12.6 | 87.4 | | 0.2 | | 99.8 |
| **Average** | | **10.2** | **89.8** | | **0.2** | | **99.8** |
| Standard deviation | | 2.2 | 2.2 | | 0.2 | | 0.2 |
| **North America combination of tumble dryer sheet with liquid anti-wrinkle fabric conditioner** | | | | | | | |
|  | | **Lint filter** | | | **Dryer Exhaust** | | |
| **Nil dryer sheet or anti-wrinkle fabric conditioner** | | % Polyester | % Cotton | | % Polyester | | % Cotton |
| Load 1 | | 9.9 | 90.1 | | 3.5 | | 96.5 |
| Load 2 | | 9.8 | 90.2 | | 0.7 | | 99.3 |
| Load 3 | | 9.5 | 90.5 | | 0.7 | | 99.3 |
| **Average** | | **9.7** | **90.3** | | **1.6** | | **98.4** |
| Standard deviation | | 0.2 | 0.2 | | 1.6 | | 1.6 |
| **1 mega dryer sheet + Double dose anti-wrinkle fabric conditioner** | | % Polyester | % Cotton | | % Polyester | | % Cotton |
| Load 1 | | 3.7 | 96.3 | | 3.5 | | 96.5 |
| Load 2 | | 3.9 | 96.1 | | 0.5 | | 99.5 |
| Load 3 | | 3.4 | 96.6 | | 0.4 | | 99.6 |
| **Average** | | **3.7** | **96.3** | | **1.4** | | **98.6** |
| Standard deviation | | 0.3 | 0.3 | | 1.8 | | 1.8 |
| **Impact of lint filter pore size** | | | | | | | |
|  | | **Lint filter** | | | **Dryer Exhaust** | | |
| **Coarse pore size lint filter** | | % Polyester | % Cotton | | % Polyester | | % Cotton |
| Load 1 | | 7.3 | 92.7 | | 2.6 | | 97.4 |
| Load 2 | | 7.1 | 92.9 | | 1.8 | | 98.2 |
| Load 3 | | 6.6 | 93.4 | | 0.8 | | 99.2 |
| **Average** | | **7.0** | **93.0** | | **1.7** | | **98.3** |
| Standard deviation | | 0.4 | 0.4 | | 0.9 | | 0.9 |
| **Fine pore size lint filter** | | % Polyester | % Cotton | | % Polyester | | % Cotton |
| Load 1 | | 6.2 | 93.8 | | 4.4 | | 95.6 |
| Load 2 | | 7.5 | 92.5 | | 0.4 | | 99.6 |
| Load 3 | | 7.2 | 92.8 | | 7.0 | | 93.0 |
| **Average** | | **7.0** | **93.0** | | **4.0** | | **96.0** |
| Standard deviation | | 0.7 | 0.7 | | 3.3 | | 3.3 |
