# Supplementary material for: The impact of fabric conditioning products and lint filter pore size on airborne microfiber pollution arising from tumble drying
Source: PLoS One. 2022 Apr 6;17(4):e0265912. doi: 10.1371/journal.pone.0265912 (PMC8985936; doi:10.1371/journal.pone.0265912)
Supplement: S7 Table — Sheet with liquid anti-wrinkle fabric conditioner. The table shows measured mass of the wash load used (kg) and microfibers collected (mg) for microfibers collected on the dryer lint filter (cycles 1–4) and released from dryer exhaust (cycles 1–4). These data are used to calculate quantity of microfibers at both of these stages in terms of ppm (parts per million, i.e. mg microfiber released per kg dry wash load). (DOCX) [file pone.0265912.s007.docx]

**S7** **Table. Microfiber release data – North America combination of tumble dryer. sheet with liquid anti-wrinkle fabric conditioner.** The table shows measured mass of the wash load used (kg) and microfibers collected (mg) for microfibers collected on the dryer lint filter (cycles 1-4) and released from dryer exhaust (cycles 1-4). These data are used to calculate quantity of microfibers at both of these stages in terms of ppm (parts per million, i.e. mg microfiber released per kg dry wash load).

| **Nil dryer sheet or anti-wrinkle fabric conditioner** | | | | | |
| --- | --- | --- | --- | --- | --- |
|  | **Load mass**  **(kg)** | **Microfiber mass**  **(mg)** | | **Microfiber release**  **(ppm)*** | |
|  |  | Lint filter | Dryer Exhaust | Lint filter | Dryer Exhaust |
| Cycle 1 – Load 1 | 2.940 | 502.2 | 129.3 | 170.83 | 43.98 |
| Cycle 1 – Load 2 | 2.940 | 430.2 | 218.1 | 146.34 | 74.20 |
| Cycle 1 – Load 3 | 2.920 | 499.2 | 160.3 | 170.96 | 54.90 |
| **Cycle 1 - Mean** | **2.933** | **477.2** | **169.2** | **162.71** | **57.69** |
| **Cycle 1 – Std Dev** | **0.012** | **40.7** | **45.1** | **14.18** | **15.30** |
| Cycle 2 – Load 1 | 2.940 | 276.6 | 117.3 | 94.08 | 39.90 |
| Cycle 2 – Load 2 | 2.940 | 335.5 | 129.7 | 114.12 | 44.12 |
| Cycle 2 – Load 3 | 2.920 | 162.2 | 150.8 | 55.54 | 51.64 |
| **Cycle 2 - Mean** | **2.933** | **258.1** | **132.6** | **87.91** | **47.22** |
| **Cycle 2 – Std Dev** | **0.012** | **88.1** | **16.9** | **29.77** | **5.95** |
| Cycle 3 – Load 1 | 2.940 | 252 | 86.5 | 85.71 | 29.41 |
| Cycle 3 – Load 2 | 2.940 | 242 | 104 | 82.31 | 35.36 |
| Cycle 3 – Load 3 | 2.920 | 277.1 | 62.4 | 94.88 | 21.36 |
| **Cycle 3 - Mean** | **2.933** | **257.0** | **84.3** | **87.63** | **28.71** |
| **Cycle 3 – Std Dev** | **0.012** | **18.1** | **20.9** | **6.50** | **7.03** |
| Cycle 4 – Load 1 | 2.940 | 192.4 | 86.5 | 65.43 | 29.44 |
| Cycle 4 – Load 2 | 2.940 | 179.2 | 79.2 | 60.96 | 26.93 |
| Cycle 4 – Load 3 | 2.920 | 161.2 | 92.1 | 55.2 | 31.54 |
| **Cycle 4 - Mean** | **2.933** | **177.6** | **85.93** | **60.53** | **29.30** |
| **Cycle 4 – Std Dev** | **0.012** | **15.7** | **6.5** | **5.19** | **2.31** |
| **1 mega dryer sheet + Double dose anti-wrinkle fabric conditioner** | | | | | |
|  | **Load mass**  **(kg)** | **Microfiber mass**  **(mg)** | | **Microfiber release**  **(ppm)*** | |
|  |  | Lint filter | Dryer Exhaust | Lint filter | Dryer Exhaust |
| Cycle 1 – Load 1 | 2.940 | 524.5 | 54.5 | 178.4 | 18.55 |
| Cycle 1 – Load 2 | 2.940 | 437.5 | 143.2 | 148.81 | 48.7 |
| Cycle 1 – Load 3 | 2.940 | 510.1 | 100.1 | 173.52 | 34.04 |
| **Cycle 1 - Mean** | **2.940** | **490.7** | **99.3** | **166.91** | **33.76** |
| **Cycle 1 – Std Dev** | **-** | **46.6** | **44.4** | **15.86** | **15.07** |
| Cycle 2 – Load 1 | 2.940 | 275 | 75.3 | 93.53 | 25.62 |
| Cycle 2 – Load 2 | 2.940 | 284.4 | 78.4 | 96.74 | 26.67 |
| Cycle 2 – Load 3 | 2.940 | 279.3 | 61.5 | 94.99 | 20.9 |
| **Cycle 2 - Mean** | **2.940** | **279.6** | **71.7** | **95.09** | **24.40** |
| **Cycle 2 – Std Dev** | **-** | **4.7** | **9.0** | **1.61** | **3.07** |
| Cycle 3 – Load 1 | 2.940 | 256.7 | 47.4 | 87.3 | 16.12 |
| Cycle 3 – Load 2 | 2.940 | 274.1 | 54.0 | 93.25 | 18.37 |
| Cycle 3 – Load 3 | 2.940 | 262.5 | 49.0 | 89.28 | 16.68 |
| **Cycle 3 - Mean** | **2.940** | **264.4** | **50.1** | **89.94** | **17.06** |
| **Cycle 3 – Std Dev** | **-** | **8.9** | **3.4** | **3.03** | **1.17** |
| Cycle 4 – Load 1 | 2.940 | 121.2 | 37.7 | 41.23 | 12.83 |
| Cycle 4 – Load 2 | 2.940 | 118.5 | 37.7 | 40.3 | 12.83 |
| Cycle 4 – Load 3 | 2.940 | 146.4 | 43.5 | 49.79 | 14.79 |
| **Cycle 4 - Mean** | **2.940** | **128.7** | **39.6** | **43.77** | **13.48** |
| **Cycle 4 – Std Dev** | **-** | **15.4** | **3.3** | **5.23** | **1.13** |

***Microfiber release (ppm) = Microfiber mass (mg) / Load mass (kg)**
