# Supplementary material for: The impact of fabric conditioning products and lint filter pore size on airborne microfiber pollution arising from tumble drying
Source: PLoS One. 2022 Apr 6;17(4):e0265912. doi: 10.1371/journal.pone.0265912 (PMC8985936; doi:10.1371/journal.pone.0265912)
Supplement: S9 Table — The table shows the number of fibers counted within 1 cm2 windows tape-lifted from the 100% Cotton and 100% Polyester T-shirts used in the testing. (DOCX) [file pone.0265912.s009.docx]

**S9 Table: Tape lift sheddability data for the tested garments.** The table shows the number of fibers counted within 1 cm^2^ windows tape-lifted from the 100% Cotton and 100% Polyester T-shirts used in the testing.

|  | **Fibers Counted on Selected Window/ cm^2^** | |
| --- | --- | --- |
| **Selected Window** | **Red Cotton T-shirt** | **Black Polyester Jersy** |
| Tape lift 1 | 4 | 2 |
| Tape lift 2 | 10 | 0 |
| Tape lift 3 | 25 | 0 |
| Tape lift 4 | 26 | 1 |
| Tape lift 5 | 17 | 1 |
| Tape lift 6 | 25 | 1 |
| **Average** | **17.8** | **0.83** |
| **Standard Deviation** | **9.20** | **0.75** |
