# Supplementary material for: The impact of fabric conditioning products and lint filter pore size on airborne microfiber pollution arising from tumble drying
Source: PLoS One. 2022 Apr 6;17(4):e0265912. doi: 10.1371/journal.pone.0265912 (PMC8985936; doi:10.1371/journal.pone.0265912)
Supplement: S10 Table — The table shows the fiber length and width of fibers collected by tape lifting from the 100% Cotton and 100% Polyester T-shirts used in the testing. (DOCX) [file pone.0265912.s010.docx]

**S10 Table: Fiber length and width data for fibers collected by tape lift.** The table shows the fiber length and width of fibers collected by tape lifting from the 100% Cotton and 100% Polyester T-shirts used in the testing.

|  | **Cotton** | | **Polyester** | |
| --- | --- | --- | --- | --- |
|  | **Length (mm)** | **Width (μm)** | **Length (mm)** | **Width (μm)** |
| Fiber 1 | 8.84 | 16.5 | 0.73 | 11.67 |
| Fiber 2 | 7.35 | 19.26 | 0.65 | 11.81 |
| Fiber 3 | 4.78 | 20.87 | 0.66 | 11.67 |
| Fiber 4 | 4.05 | 35.25 | 0.37 | 13.43 |
| Fiber 5 | 2.45 | 18.99 | 0.86 | 10.76 |
| Fiber 6 | 1.9 | 21.03 | 0.59 | 13.05 |
| Fiber 7 | 1.87 | 20.87 | 1.00 | 11.67 |
| Fiber 8 | 4.53 | 33.00 | 0.69 | 11.22 |
| Fiber 9 | 1.34 | 19.26 | 0.63 | 11.67 |
| Fiber 10 | 0.99 | 29.75 | 0.53 | 13.30 |
| Fiber 11 | 4.29 | 18.54 | 3.18 | 16.60 |
| Fiber 12 | 5.22 | 20.29 | 0.70 | 11.67 |
| Fiber 13 | 3.31 | 20.87 | 0.81 | 14.41 |
| Fiber 14 | 1.42 | 19.87 | 10.78 | 11.22 |
| Fiber 15 | 4.11 | 17.01 | 0.54 | 14.76 |
| Fiber 16 | 2.96 | 21.51 | 1.05 | 13.03 |
| Fiber 17 | 2.12 | 36.63 | 1.29 | 11.81 |
| Fiber 18 | 4.82 | 19.61 | 0.75 | 15.76 |
| Fiber 19 | 2.67 | 25.70 | 0.72 | 11.81 |
| Fiber 20 | 6.65 | 26.41 | 1.56 | 11.07 |
| **Average** | **3.78** | **23.06** | **1.40** | **12.62** |
| **Standard Deviation** | **2.11** | **6.03** | **2.29** | **1.64** |
